# A Seven-Marker Signature and Clinical Outcome in Malignant Melanoma: A Large-Scale Tissue-Microarray Study with Two Independent Patient Cohorts

**DOI:** 10.1371/journal.pone.0038222

**Published:** 2012-06-07

**Authors:** Stefanie Meyer, Thomas J. Fuchs, Anja K. Bosserhoff, Ferdinand Hofstädter, Armin Pauer, Volker Roth, Joachim M. Buhmann, Ingrid Moll, Nikos Anagnostou, Johanna M. Brandner, Kristian Ikenberg, Holger Moch, Michael Landthaler, Thomas Vogt, Peter J. Wild

**Affiliations:** 1 Department of Dermatology, University Hospital of Regensburg, Regensburg, Germany; 2 Department of Computer Science, ETH Zurich, Zurich, Switzerland; 3 Institute of Pathology, University Hospital of Regensburg, Regensburg, Germany; 4 Central Tumor Registry Regensburg, Regensburg, Germany; 5 Department of Computer Science, University of Basel, Basel, Switzerland; 6 Competence Center for Systems Physiology and Metabolic Diseases, Institute of Cell Biology, Zurich, Switzerland; 7 Department of Dermatology, University Hospital of Hamburg-Eppendorf, Hamburg, Germany; 8 Institute of Surgical Pathology, University Hospital Zurich, Zurich, Switzerland; 9 Department of Dermatology, Saarland University Hospital, Homburg/Saar, Germany; The University of Queensland, Australia

## Abstract

**Background:**

Current staging methods such as tumor thickness, ulceration and invasion of the sentinel node are known to be prognostic parameters in patients with malignant melanoma (MM). However, predictive molecular marker profiles for risk stratification and therapy optimization are not yet available for routine clinical assessment.

**Methods and Findings:**

Using tissue microarrays, we retrospectively analyzed samples from 364 patients with primary MM. We investigated a panel of 70 immunohistochemical (IHC) antibodies for cell cycle, apoptosis, DNA mismatch repair, differentiation, proliferation, cell adhesion, signaling and metabolism. A marker selection procedure based on univariate Cox regression and multiple testing correction was employed to correlate the IHC expression data with the clinical follow-up (overall and recurrence-free survival). The model was thoroughly evaluated with two different cross validation experiments, a permutation test and a multivariate Cox regression analysis. In addition, the predictive power of the identified marker signature was validated on a second independent external test cohort (n = 225). A signature of seven biomarkers (Bax, Bcl-X, PTEN, COX-2, loss of β-Catenin, loss of MTAP, and presence of CD20 positive B-lymphocytes) was found to be an independent negative predictor for overall and recurrence-free survival in patients with MM. The seven-marker signature could also predict a high risk of disease recurrence in patients with localized primary MM stage pT1-2 (tumor thickness ≤2.00 mm). In particular, three of these markers (MTAP, COX-2, Bcl-X) were shown to offer direct therapeutic implications.

**Conclusions:**

The seven-marker signature might serve as a prognostic tool enabling physicians to selectively triage, at the time of diagnosis, the subset of high recurrence risk stage I–II patients for adjuvant therapy. Selective treatment of those patients that are more likely to develop distant metastatic disease could potentially lower the burden of untreatable metastatic melanoma and revolutionize the therapeutic management of MM.

## Introduction

Cutaneous malignant melanoma (MM), represents the most common cause of death from skin cancer, and, apart from female lung cancer, it is the tumor entity with the highest increase in incidence worldwide [1]. MM is characterized by a multi-factorial etiology. Sun exposure and genetic susceptibility have been proposed as major etiological and predisposing factors and may explain the reported increase of incidence to some degree [2].

De facto, the prognosis of patients with MM may only be conditionally derived from clinical and histological parameters. According to the AJCC 2009 classification [3], the findings of vertical tumor thickness [4], tumor ulceration [5], and sentinel node biopsy [6] represent the most dominant prognostic factors. In stage pT1 melanomas (≤1.00 thickness), the mitotic rate (histologically defined as mitoses/mm^2^) has to be considered as additional prognostic parameter [3].

In MM, multiple cellular factors are known to be deregulated in the initiation and progression phase of the tumor; among these are protein regulators of the cell cycle, apoptosis, signal transduction, cell adhesion and matrix digestion. A plethora of single biomarkers have been evaluated for outcome prediction in melanoma patients; e.g. Weinlich and coworkers identified metallothionein expression as an independent negative marker of melanoma progression in thin primary tumours [7]. Despite the fact that hundreds of such studies sought to assess the potential prognostic value of molecular markers in predicting the course of cutaneous MM, there are only two prognostic models with potential for translation into the clinic. Gould-Rothberg et al. [8] published a genetic-algorithm based five-marker solution, and Kashani-Sabet et al. [9] reported a three-marker model. However, no predictive molecular profiles for therapy optimization applicable for routine clinical assessment of MM are available, according to the latest review meta-analyses [10], [11].

To this end, we examined the immunohistochemical (IHC) expression of 70 candidate biomarkers of MM including regulating proteins of the cell cycle and apoptosis control, factors of signal-transduction, cell adhesion, transcription-factors, differentiation, and melanoma-specific antigens using tissue microarrays (TMAs). The study was based on extensive follow-up investigation of a total of 589 patients with primary MM from two independent cohorts, and was initiated to identify a clear set of reliable IHC markers for routine clinical assessment of patients with primary MM. Accordingly, this biomarker study aimed at identifying an independent prediction model for clinical outcome in patients with MM.

## Materials and Methods

### Ethics Statement

The study for both cohorts was approved by the local scientific ethics committees (approval no.: 07/093 for Regensburg and MC-028/08 for Hamburg).

### Tissue Microarrays (TMAs)

TMAs were constructed as described previously [12] and based on primary melanoma material, collected between 1994 and 2006. TMA 1, the primary cohort, contained single tissue punch samples from 364 consecutive (non-selected), formalin-fixed, paraffin-embedded MMs of 364 different patients and were from the Department of Dermatology, University Hospital of Regensburg, Germany. TMA 2, the secondary cohort (independent external validation cohort), consisted of consecutive (non-selected) MM samples from 235 patients of the Department of Dermatology, University Hospital Hamburg-Eppendorf, Germany. TMA 2 contained two tissue cores per melanoma specimen. For patients with multiple subsequent neoplasms, only initial and single primary MMs were included. H&E-stained slides of all MMs were evaluated by two histopathologists (TV, PJW). The clinicopathological characteristics of the two independent cohorts of melanoma patients are given in Table S1. Clinical follow-up data, provided by the local tumor registries, were available for all patients of the primary cohort (n = 364) and 231 patients of the secondary cohort. Patients were censored at 120 months, if their follow-up exceeded the 10-year scope of the study. The retrospective study implemented the REMARK guidelines [13].

### Selection of Candidate Biomarkers

The primary antibodies used in this study were selected for reporting on key aspects of apoptosis, cell cycle, signal transduction, cell adhesion, differentiation and proliferation, and tumor metabolism (Table S2). The candidate markers were chosen because of their described role in MM in the literature [10], [11], [14] or on the basis of previous studies by our group [12], [15].

### Immunohistochemical Analysis

Paraffin-embedded preparations of melanoma tissues were screened for protein expression according to standardized IHC protocols as described previously [12], [15]. Immunohistochemical stainings were performed for 70 different primary antibodies (source and concentrations are listed in Table S2). Negative controls were obtained by omitting the primary antibody. Specificity of commercial antibodies of the seven-marker signature has been thoroughly tested by immunoblotting (Figures S1), immunohistochemical analysis of melanocytes and melanoma cell lines using a cell pellet microarray (Figure S2), and immunostaining of whole melanoma sections (Figures S3A–D). Nocito et al. [16] have already shown that intra-tumour heterogeneity does not significantly affect the ability to detect clinico-pathological correlations on tissue microarrays (TMAs), probably because of the large number of tumors that can be included in TMA studies. Figures S3A–D show whole tissue sections of primary malignant melanomas stained for our seven-marker signature. Only Bax and MTAP immunoreactivity was patchy in some cases (Figure S3A) but homogeneous in most of the remaining ones (S3B–D).

Two dermatohistopathologists (SM, ML) performed a blinded evaluation of the stained slides. In case of discordant scoring results a consensus score was assigned. Cytoplasmic and nuclear immunoreactivity were evaluated using a stepwise scoring system (0 to 4+): 0 (negative): no cytoplasmic staining or 0% of cell nuclei stained; 1+: weak cytoplasmic staining or less than 20% of cell nuclei stained; 2+: moderate cytoplasmic staining or 21 to 50% of cell nuclei stained; 3+: strong cytoplasmic staining or 51 to 90% of cell nuclei stained; 4+: very strong cytoplasmic staining or nuclear staining greater than 90%. This semiquantitative scoring system was consistently used for all 70 markers analyzed. TMA spots with a lack of tumor tissue or presence of necrosis or crush artifact were excluded from the analysis. For the validation cohort (TMA 2), a single score was assigned, taking the strongest immunoreactivity of the two spots into account.

**Table 1:** Univariate Cox proportional hazard regeression models.

No.	Variable	Coefficients	Std. Error	P-Value	Hazard ratio	95% CI	
1	AKT3	0.188	0.164	0.252	1.206	0.875	1.663
2	BAX	0.441	0.091	<0.001	1.554	1.301	1.857
3	BCL2	−0.098	0.087	0.260	0.907	0.765	1.075
4	BCL2L1	0.092	0.086	0.285	1.096	0.926	1.298
5	Bcl-X	0.391	0.138	0.005	1.479	1.128	1.940
6	BMI1	−0.043	0.104	0.677	0.958	0.781	1.174
7	B-Raf	0.123	0.081	0.130	1.130	0.965	1.325
8	Caveolin	−0.028	0.114	0.806	0.972	0.777	1.217
9	CD166	0.089	0.173	0.606	1.094	0.778	1.536
10	CD20	0.547	0.188	0.004	1.728	1.195	2.498
11	CD44	−0.154	0.107	0.150	0.857	0.695	1.057
12	CDK2	0.146	0.077	0.057	1.158	0.996	1.345
13	c-Kit	−0.119	0.068	0.082	0.888	0.776	1.015
14	c-Myc	0.044	0.075	0.558	1.045	0.903	1.209
15	COX-2	0.297	0.123	0.016	1.345	1.058	1.710
16	CTNNB1	−0.340	0.108	0.002	0.712	0.577	0.879
17	CXCR4	−0.025	0.084	0.763	0.975	0.827	1.150
18	CyclinA	0.261	0.350	0.456	1.298	0.654	2.575
19	Cyclin-D1	0.174	0.091	0.057	1.190	0.995	1.424
20	E-Cadherin	−0.118	0.068	0.081	0.889	0.778	1.015
21	ephB2	0.054	0.119	0.648	1.056	0.836	1.333
22	ephrinB2	0.005	0.120	0.968	1.005	0.794	1.272
23	Ezrin	−0.074	0.080	0.357	0.929	0.795	1.086
24	FAS	0.103	0.126	0.410	1.109	0.867	1.418
25	FZD7	0.232	0.108	0.032	1.262	1.020	1.560
26	Glut-1	0.042	0.088	0.636	1.043	0.877	1.240
27	HIF1A	0.117	0.112	0.297	1.124	0.902	1.400
28	HMB 45	0.038	0.081	0.638	1.039	0.886	1.218
29	IGF2	−0.168	0.100	0.093	0.845	0.695	1.029
30	iNOS	0.149	0.081	0.067	1.160	0.990	1.360
31	ITGA4 (CD49d)	0.407	0.096	<0.001	1.502	1.245	1.812
32	Ki-67	0.046	0.137	0.735	1.047	0.801	1.370
33	L1CAM	−0.005	0.104	0.961	0.995	0.811	1.220
34	Melan A	0.082	0.087	0.344	1.085	0.916	1.286
35	Melanin	0.112	0.080	0.160	1.119	0.957	1.308
36	MHCI	−0.080	0.108	0.461	0.923	0.747	1.142
37	MITF	−0.127	0.083	0.124	0.881	0.749	1.036
38	MLH1	0.254	0.088	0.004	1.290	1.086	1.531
39	MSH2	0.055	0.102	0.588	1.057	0.865	1.291
40	MTAP	−0.621	0.231	0.007	0.537	0.342	0.845
41	MTSS1	−0.016	0.148	0.913	0.984	0.736	1.316
42	MUM1p	0.053	0.090	0.559	1.054	0.884	1.257
43	NFKB	−0.083	0.085	0.329	0.920	0.779	1.087
44	NRAS	0.206	0.143	0.151	1.228	0.928	1.626
45	p14	0.039	0.115	0.730	1.040	0.831	1.302
46	p15	−0.187	0.111	0.092	0.829	0.667	1.031
47	p16	−0.051	0.083	0.540	0.950	0.808	1.118
48	p21	0.134	0.084	0.110	1.143	0.970	1.347
49	p27	−0.091	0.097	0.347	0.913	0.756	1.104
50	p53	0.141	0.073	0.054	1.152	0.998	1.330
51	p75 (NGFR)	0.100	0.116	0.386	1.105	0.881	1.386
52	P-Cadherin	−0.104	0.096	0.279	0.902	0.747	1.088
53	PGF	0.031	0.066	0.646	1.031	0.905	1.175
54	Phospho-Akt (Thr308)	0.134	0.096	0.161	1.144	0.948	1.380
55	Phospho-CTNNB1 (Ser33/37/Thr41)	0.016	0.081	0.842	1.016	0.867	1.191
56	Phospho-Rb (Ser807/811)	−0.138	0.293	0.637	0.871	0.491	1.546
57	Phospho-Stat1 (Ser727)	0.107	0.098	0.275	1.113	0.918	1.348
58	PMP2	0.078	0.099	0.430	1.081	0.890	1.313
59	PPARA	−0.147	0.098	0.135	0.863	0.712	1.047
60	PTEN	0.272	0.102	0.008	1.312	1.075	1.601
61	Rb	0.233	0.156	0.135	1.263	0.930	1.714
62	Ro52	−0.187	0.234	0.425	0.830	0.525	1.312
63	S1P1	0.049	0.079	0.533	1.051	0.899	1.228
64	SKP2	0.047	0.085	0.580	1.048	0.887	1.238
65	Stat 1	0.010	0.114	0.928	1.010	0.808	1.263
66	Survivin	−0.104	0.094	0.269	0.901	0.749	1.084
67	TGFB1	−0.118	0.108	0.276	0.889	0.719	1.099
68	TOP2A	−0.205	0.092	0.026	0.814	0.680	0.975
69	VEGFR2	−0.048	0.097	0.619	0.953	0.788	1.153
70	XIAP	0.037	0.088	0.673	1.038	0.874	1.233

Bold face representing variables with p-values <0.05.

CI, confidence interval.

### Statistical Analysis

P-values lower than 0.05 were considered to indicate statistical significance. Statistical analyses were conducted using R version 2.11. A biomarker signature for prognosis of patients with melanoma was developed and validated in three major steps:

#### A) Model Discovery

First, the prognostic value for each of the of the 70 markers that met all quality-control steps was assayed by univariate Cox proportional hazards regarding overall survival, and those markers reaching significance at p = 0.05 with a false-discovery rate of 0.15 were included in subsequent model building. A risk score was calculated for each patient by a linear combination of the univariate Cox regression coefficients and the corresponding IHC measurements 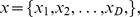 where *D* is the number of markers in the signature. Single can be not available due to missing TMA spots. Finally, the score is normalized by the number of markers measured:
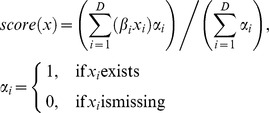



Based on this risk score, patients were assigned to a high risk group and a low risk group, split at the 50^th^ percentile (median) of all scores. Thus, the final model consists of the coefficient vector β****** and the median threshold θ. Nonparametric Kaplan-Meier estimators [17] were used to analyze overall survival and recurrence-free survival. Differences between survival estimates were assessed with the log-rank test (LRT) [18]. Finally, a multivariable Cox regression model was adjusted, testing the independent prognostic relevance of our risk score. Besides the proposed biomarker signature, age, gender, tumor thickness, Clark level, and nodal status were included in the Cox model as covariates.

**Figure 1:**
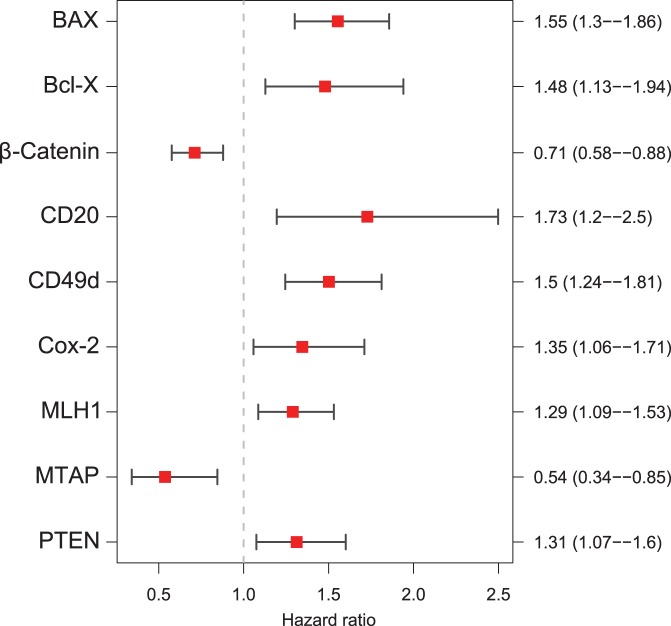
Hazard Ratios of the Nine-Marker Signature learned by the FDR selection procedure. Markers with a hazard ratio smaller than 1.00 represent “protective markers” (MTAP, β-Catenin). Those with hazard ratios larger than 1.00 represent “risk markers” (Bax, Bcl-X, infiltration with CD20 positive B-lymphocytes, CD49d, COX-2, MLH1 and PTEN).

#### B) Internal Model Validation

The validity of the learning procedure and hence the accuracy of the signature was assessed in three different validation experiments. The cross validation experiments were conducted as follows:

1. Divide the patients into K cross-validation folds (groups) at random.
2. For each fold k  = 1, 2, …, K.
  - Find a subset of univariate statistical significant (LRT p<0.05) predictors for the overall survival, using all of the patients except those in fold k.
  - Filter the selected predictors based on a false discovery rate (FDR) of 0.15.
  - Using just this subset of predictors, build a multivariate linear model, using all of the patients except those in fold k.
  - Use the model to predict the scores for the patients in fold k.
3. Aggregate the out-of-bag predictions of all patients and split them in two groups based on the median predicted score.
4. Calculate the Kaplan-Meier estimator for each group and report the LRT p-value of their difference in survival expectation.

First, leave-one-out cross validation was employed by excluding one patient at a time from the training set and subsequently scoring the left out patient with the signature learned from the rest of the patients. Repeating this procedure 364 times yields a leave-one-out score estimate for each patient in the study. Second, 10-fold cross validation was conducted by partitioning the dataset into 10 parts of equal size using 90% of the patients for learning and 10% for validation. The procedure was repeated 10 times resulting in a 10-fold cross validation score for each patient. The third validation experiment was conducted to assess, if the proposed marker selection procedure is prone to over fitting. To this end, the target variable was randomly permuted and a model was learned to predict the risk score based on this distorted data.

**Figure 2:**
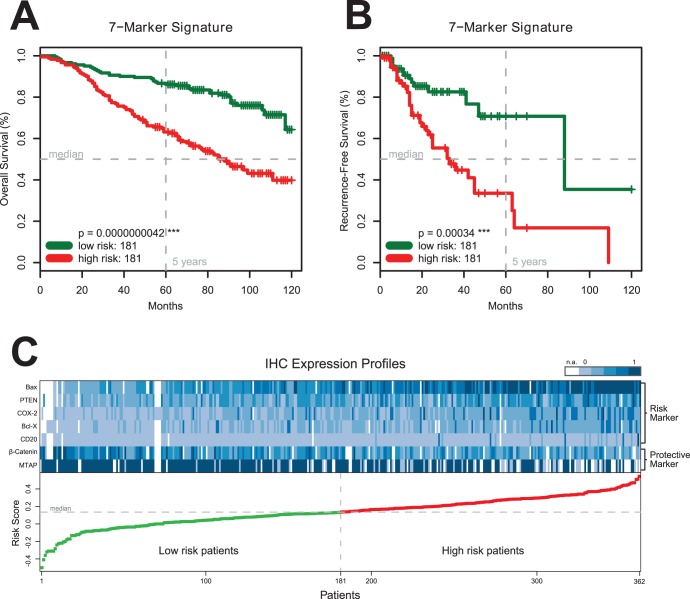
The Seven-Marker Signature and Survival of 362 Patients with Primary MM. Panels **A** and **B** show Kaplan-Meier estimates of overall and recurrence-free survival for high risk patients (red) and low risk patients (green) from the primary cohort according to the final seven-marker signature (Panels A, B). Equality in survival expectance of the subgroups is assessed by the log-rank test. The difference between high risk patients and low risk patients is highly significant (p<0.001) for the seven-marker signature. Panel **C** shows the IHC expression profiles of 362 tumor specimens from the primary cohort ordered by their predicted risk score. Each column represents an individual patient consisting of the expression values of the seven-marker signature (5 risk markers and 2 protective markers). The magnitude of the corresponding risk score is plotted below for 181 low risk patients (green) and 181 high risk patients (red). IHC expression values were scaled between 0 (light blue) and 1 (dark blue) for plotting only. White cells represent missing values (n.a.).

**Table 2:** Clinical characteristics of the primary cohort of patients with MM (TMA 1).

	High risk (N = 181)	Low risk (N = 181)	p-Value: high vs. low risk	Hazard ratio (95% CI) #3	p-Value #3
**7-Marker risk score**	0.267±0.092	0.0017±0.12	<<0.0001#1	5.1 (1.4–18.2)	0.012*
**Age – yr**	59.5±15.0	57.7±14.9	0.263#1	1.03 (1.02–1.05)	0.000011***
**Tumor thickness – mm**	2.52±2.38	1.40±2.21	0.00000646#1	1.05 (0.97–1.14)	0.24
**Clark level**	3.66±0.739	2.93±0.83	<<0.0001***	1.8 (1.4–2.5)	0.000098***
**Sex – no. of patients (%)**					
Male	105 (58)	89 (49.2)	1#2	1.9 (1.3–2.8)	0.0019**
Female	76 (42)	92 (50.8)			
**Nodal status (%)**					
N0	158 (90.8)	159 (95.8)	0.084#2	1.6 (0.8–3.2)	0.15
N1-N3	16 (9.2)	7 (4.22)			

#1 Welch two sample t-test,

#2 Fisher’s exact test,

#3 Multivariate Cox regression,

*** p-Value <0.001.

Comparing high-risk patients (first column) with low-risk patients (second column) based on their seven-marker risk score showed a significant difference in tumor thickness (p<0.001) and Clark levels (p<0.001), and no difference in nodal status (p = 0.084), sex (p = 1) and age (p = 0.263). Furthermore, hazard ratios and p-values were reported for a multivariate Cox regression model comprising all listed variables. Regarding overall survival the seven-marker risk score was statistically significant (p<0.05) independent of sex, age, nodal status, clark level and tumor thickness. Continuous variables are reported with mean and standard deviation and categorical variables are listed with number of counts and percentages.

**Figure 3:**
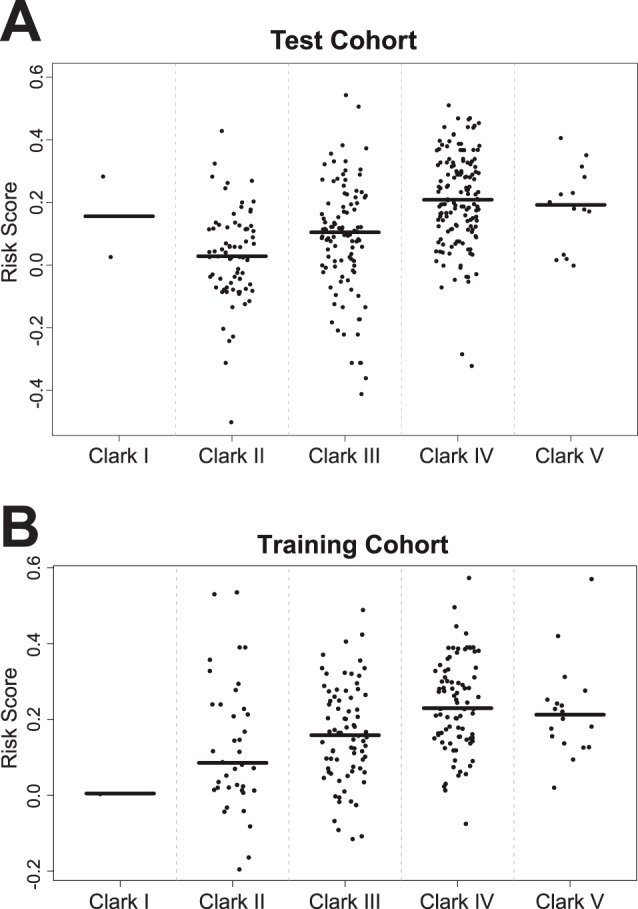
Dot blots of risk scores for different Clark levels in the training (Panel A) and testing (Panel B) cohort. Horizontal lines represent median risk scores for each subgroup.

**Figure 4:**
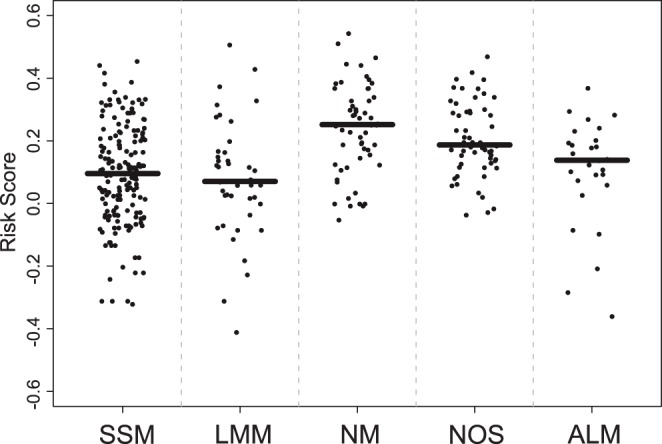
Dot blot of risk scores for the various histological subtypes of melanoma as classified by the ICD-10 (International Statistical Classification of Diseases and Related Health Problems, 10th revision). SSM, superficial spreading melanoma; LMM, lentigo maligna melanoma; NM, nodular melanoma; NOS, not otherwise specificed; ALM, acral lentiginous melanoma. Horizontal lines represent median risk scores for each subgroup.The aim of this study was to provide a maximum of prognostic and therapeutically relevant information by a minimum of markers combined in a clear signature. For the sake of clinical feasibility and cost saving, an IHC marker set suitable for routine clinical assessment should be based on a limited number of antibodies. Accordingly, the nine-marker signature was reduced by the risk marker with the lowest Cox regression coefficient β, i.e. MLH1 (β = 0.254).

**Figure 5:**
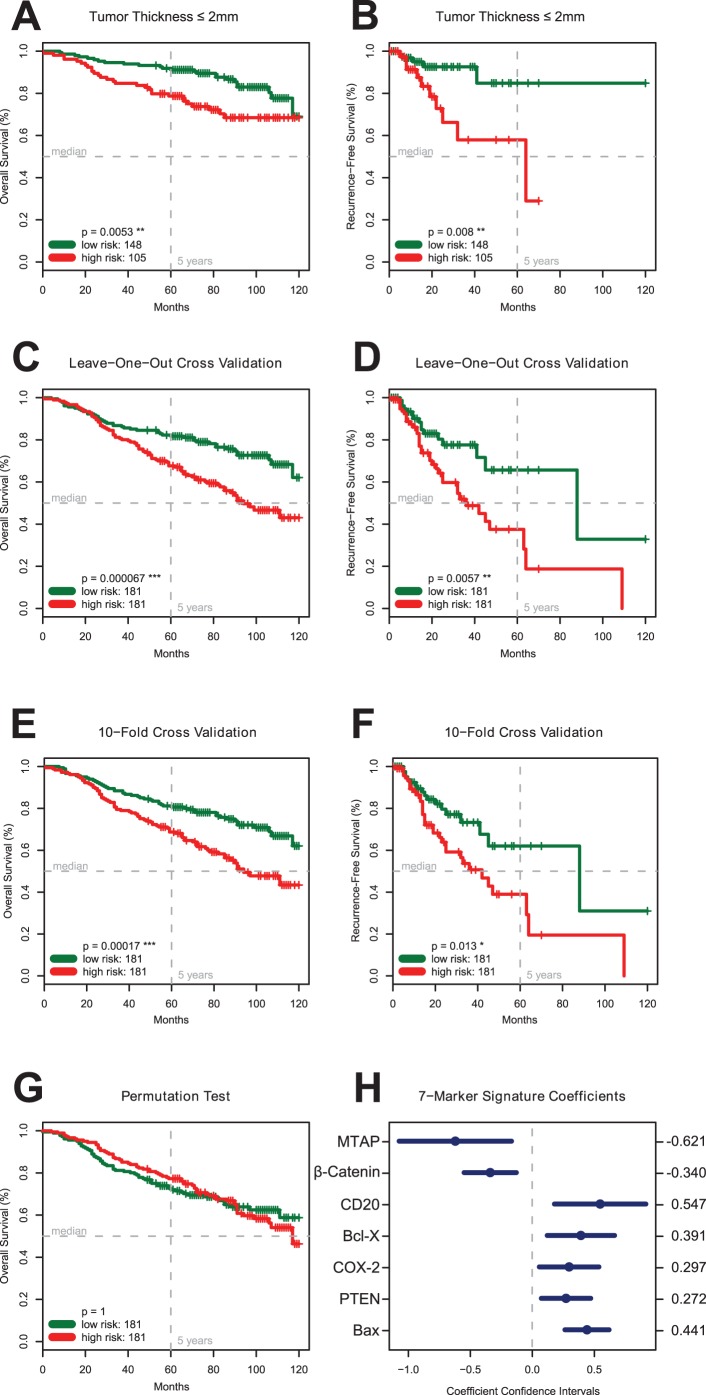
Statistical Analyses. Panel A, B. The Seven-Marker Signature and Survival of Patients with a Tumor Thickness ≤2.0 mm (TMA 1). Kaplan-Meier estimates show a significantly lower overall (p<0.01, Panel A) and recurrence-free survival (p<0.01, Panel B) for patients with a comparatively low tumor thickness ≤2.0 mm but high-risk score. **Panel C, D. Leave-One-Out Cross Validation.** To investigate the generalization error of the models produced by the FDR signature learning procedure a leave-one-out cross validation experiment was conducted on the primary cohort of 362 MM patients. The resulting risk score could significantly (p<0.001) differentiate between patients with higher or lower overall survival expectance. The two patient groups also significantly (p<0.01) differ in recurrence-free survival. **Panel E, F. 10-Fold Cross Validation.** In addition, the FDR marker selection procedure was tested by a 10-fold cross validation experiment on the 362 patients of the primary cohort (TMA 1) resulting in still significant estimates for overall survival (p<0.001; Panel E) and recurrence-free survival (p<0.05; Panel F). **Panel G. Permutation Test.** In addition to the cross validation experiments a permutation test was conducted to assess if the signature learning procedure is over fitting the data set. The resulting signature, which was learned on permuted overall survival data, was not able (p = 1) to discriminate between patients with differing survival expectance. This result indicates that the proposed learning procedure does not over fit the data. **Panel H. Coefficients and Confidence Intervals of the Seven-Marker Signature.** The coefficients from the univariate Cox proportional hazard models are used in a weighted linear combination to predict the risk score for each patient. Markers with negative coefficients represent protective markers (MTAP, β-Catenin); those with positive coefficients risk markers (Bax, Bcl-X, PTEN, COX-2, and presence of CD20 positive lymphocytes,).

#### C) External Model Validation

Our marker signature was validated on an external test cohort from Hamburg, Germany. Again, a risk score was calculated for each patient by a linear combination of the univariate Cox regression coefficients β from the initial test cohort and the IHC measurements of the external cohort. Based on this risk score, patients were assigned to a high risk group and a low risk group, split at the 50^th^ percentile (median) of all scores. Finally, a multivariable Cox proportional hazards model was adjusted, testing the independent prognostic relevance of our risk score. Besides the proposed biomarker signature, age, gender, tumor thickness, Clark level, and nodal status were included in the Cox model as covariates.

**Figure 6:**
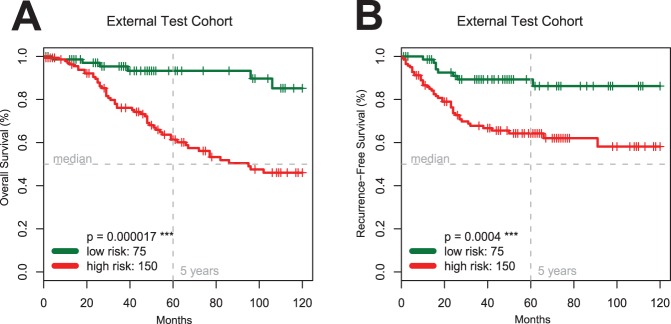
Validation of the Seven-Marker Signature and the FDR Marker Selection Procedure. Kaplan-Meier estimates of overall (Panel **A**) and recurrence-free survival (Panel **B**) for the independent external test cohort of 225 patients (TMA 2) confirm the predictive prognostic power of the signature (p<0.001).

**Table 3:** Clinical characteristics of the external test cohort of patients with MM (TMA 2).

	High risk (N = 150)	Low risk (N = 75)	p-Value: high vs. low risk	Hazard ratio (95% CI)#3	p-Value#3
**7-Marker risk score**	0.270±0.098	0.04±0.071	<<0.0001#1	14.45 (1.68–124.49)	0.015*
**Age – yr**	55.2±16	52.6±6.6	0.267#1	1.02 (1.00–1.04)	0.11
**Tumor thickness – mm**	2.55±2.67	1.08±1.17	0.0000000512#1	1.19 (1.07–1.32)	0.0017**
**Clark level**	3.58±0.814	2.97±0.87	0.0000016#1	1.81 (1.06–3.09)	0.030*
**Sex – no. of patients (%)**					
Male	81 (55.1)	41 (54.7)	1#2	1.55 (0.84–2.85)	0.16
Female	66 (44.9)	34 (45.3)			
**Nodal status (%)**					
N0	131 (93.6)	73 (97.3)	0.34#2	3.18 (1.27–7.94)	0.013*
N1–N3	9 (6.43)	2 (2.67)			

#1 Welch two sample t-test,

#2 Fisher’s exact test,

#3 Multivariate Cox regression,

* p-Value <0.05,

*** p-Value <0.001.

Comparing high-risk patients (first column) with low-risk patients (second column) based on their seven-marker risk score showed a significant difference in tumor thickness (p<0.001) and Clark levels (p<0.001), and no difference in sex (p = 1), age (p = 0.267) and nodal status (p = 0.34). Furthermore, hazard ratios and p-values were reported for a multivariate Cox regression model comprising all listed variables. Regarding overall survival the seven-marker risk score was statistically significant (p<0.05) independent of sex, age, nodal status, clark score and tumor thickness. Continuous variables are reported with mean and standard deviation and categorical variables are listed with number of counts and percentages.

## Results

### Discovery of the Seven-Marker Signature

Prognostic power of the 70 markers was assessed by calculating univariate proportional hazard models [19], yielding eleven markers significantly associated with overall survival (Table 1). To correct for multiple testing, the false discovery rate (FDR) procedure [20] was applied with a FDR of 0.15 reducing the set of significantly associated markers to nine (Figure 1) which were correlated with death from any cause: MTAP and β-Catenin were so called “protective markers”, where loss of expression was associated with worse outcome. The remaining other seven markers were assigned “risk markers”.

**Figure 7:**
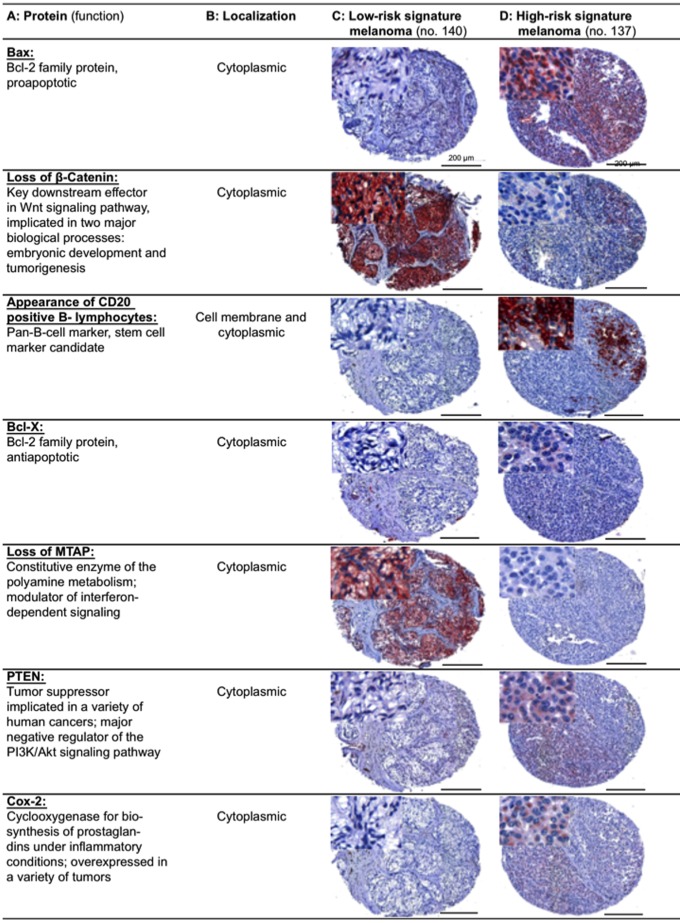
Immunohistochemically stained TMA specimens illustrating the Seven-Marker Signature for a patient with a high-risk and another patient with a low-risk melanoma. The low-risk melanoma (**Column C**) showed a strong cytoplasmic staining for β-Catenin and MTAP, respectively. Immunoreactivity of these two protective markers was not found in the high-risk melanoma (**Column D**). In contrast, the high-risk melanoma demonstrated a moderate to strong cytoplasmic staining for Bax, Bcl-X, PTEN, COX-2, and infiltration with CD20 positive B-lymphocytes.

Finally, CD49d, an α4-integrin (ITGA4) participating in cell-surface mediated signaling and adhesion, was dropped because the antibody failed our validation processes due to generation of multiple bands on western blot using a panel of melanoma cell lines and melanocytes [21] (Figure S1). Exclusion of CD49d resulted in a final seven biomarker signature. Besides CD49d, Bcl-X, MTAP, and to a lesser extent CTNNB1 and Bax also displayed multiple bands (Figure S1). However, all antibodies except CD49d showed at least one main (strongest) western blot signal.

Among the 362 patients of the primary cohort, patients with a high-risk seven-marker signature had a shorter median overall survival than the patients with a low-risk seven-marker signature (88 months versus not reached) and the difference between the two patient groups was significant (p<0.00001) (Figure 2A). The high-risk seven-marker signature was associated with a median recurrence-free survival of 33 months, whereas the low-risk seven-marker signature was associated with a median recurrence-free survival of 88 months (p<0.001) (Figure 2B). The heatmap in Figure 2C shows the IHC expression profiles of these 362 tumor specimens from the primary cohort ordered by their predicted risk score, indicating no high correlation between the selected markers. Figure S4 depicts all correlations between the markers of interest. Having the same sign, risk markers are naturally slightly correlated, but the maximal correlation between Bcl-X and COX-2 is only 0.36.

Comparing high-risk with low-risk patients (Table 2) based on their seven-marker risk score showed a significant difference in tumor thickness (p<0.001) and Clark level (p<0.001) and no difference in nodal status (p = 0.08), sex (p = 1) and age (p = 0.26). Risk scores significantly increased with increasing Clark levels (Figures 3A and 3B). Patients with nodular malignant melanomas (NM) showed the highest risk scores. Dot blots of risk scores for the various histological subtypes of malignant melanoma as classified by the ICD-10 are given in Figure 4.

According to multivariate Cox regression analysis, the seven-marker risk score, age, Clark level, and sex, were significantly associated with death from any cause among the 320 patients (113 events, 44 observations were deleted due to missing values). Table 2 summarizes the characteristics of variables that were included in the multivariate Cox model.

A subgroup analysis of 253 patients with a tumor depth of ≤2 mm revealed that those 148 patients with a high-risk marker signature had a significantly (p<0.01) shorter overall survival (Figure 5A) and recurrence-free survival (p<0.01) than the 105 patients with a low-risk marker signature (Figure 5B).

**Figure 8:**
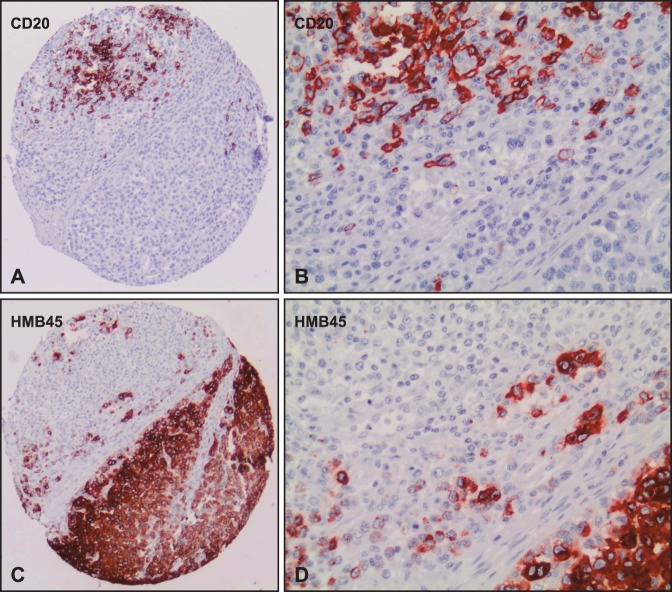
High resolution images of case no. 137 on the tissue microarray. Serial sections of the tissue microarray (TMA 1) was immunohistochemically stained with CD20 (Panel A, B) and HMB45 (Panel C, D) to show CD20 positive B-lymphocytes within and adjacent to melanoma cells (case no. 137).

**Figure 9:**
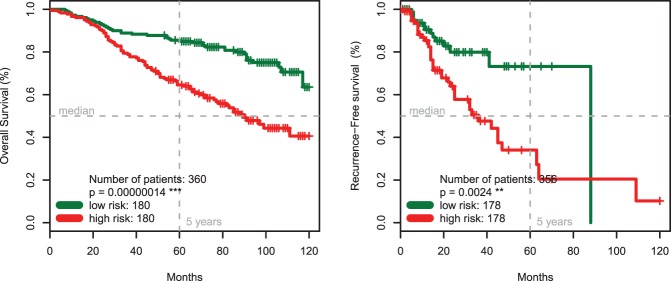
The Six-Marker Signature (without CD20) and Survival of Patients with Malignant Melanoma. Kaplan-Meier estimates show a significantly lower overall (p<0.00001, Panel A) and recurrence-free survival (p<0.01, Panel B) for melanoma patients with high-risk score.

### Internal Statistical Validation of the Seven-Marker Signature

The cross validation experiments showed comparable results and demonstrated that learning a marker signature for overall and recurrence-free survival was feasible and reproducible (Figures 5C–F). For leave-one-out cross validation, patients with high risk scores had a median survival of 94 months whereas median survival for patients with low risk signature was not reached (Figure 5C). The difference in survival expectance between patients with high-risk score and low-risk score was highly significant (p<0.0001). Although 10-fold cross validation has lower bias and higher variance, the difference between the high risk and low risk group (94 month versus not reached) was still significant (p<0.001, Figure 5E). In contrast to the cross validation experiments it was not possible to learn a signature to predict permuted labels (p>0.5), which indicates that the proposed learning procedure is not over fitting (Figure 5G). All coefficients and confidence intervals of the seven-marker signature are reported in Figure 5H.

### Model Validation of the Seven-Marker Signature on an External Test Cohort

The clinical characteristics of the 225 patients in the external test cohort are listed in Table S1. Patients with a high-risk marker signature had a significantly (p<0.0001) shorter median overall survival compared to patients with a low-risk signature (Figure 6A). Comparing high-risk patients (Table 3) with low-risk patients based on their seven-marker risk score showed a significant difference in tumor thickness (p<0.001) and Clark levels (p<0.001), but no difference in sex (p = 1), age (p = 0.27) and nodal status (p = 0.34).

According to multivariate Cox regression including the seven-marker risk score, sex, age, Clark level, nodal status, and tumor thickness, the seven-marker signature was significantly associated with overall survival (p<0.05, Table 3). Additionally, the recurrence-free survival differed significantly between the two risk groups (p<0.001; Figure 6B).

## Discussion

In this retrospective study of 364 melanoma patients, we identified an independent seven-marker signature of prognosis. Notably, the predictive power of the signature was carefully validated and confirmed on a secondary independent external test cohort (n = 225). With a total of 27,055 specimens of primary MMs analyzed by IHC, this TMA study is unmatched in the literature. An individual patient’s risk score can easily be calculated given the immunoreactivity scores for the seven markers and the estimated coefficients (Figure 5H). Figure 7 shows stained TMA specimens illustrating the Seven-Marker Signature for a patient with a high-risk and another patient with a low-risk melanoma.

One of the main statistical problems in large scale IHC studies are missing values in the design matrix due to missing or corrupt spots on the TMA. The more markers are investigated the higher the chance that at least one value is missing per patient. Frequently this problem is tackled by either sacrificing a larger number of patient records or by employing volatile multiple imputation techniques. In this study 9.3% of values were missing, reducing the set of patients with all IHC measurements from 364 to 170. Algorithms like random survival forests [22] and ensemble learning with gradient boosting [23] are capable of dealing with missing values, but lead to models that are not intuitively interpretable and difficult to implement in clinical practice. To overcome these problems we employed a learning procedure which is invariant to missing values and results in an easily interpretable and practically applicable linear model.

In early disease stages, application of an IHC based test to examine a MM patient’s tumor tissue at a molecular level suggests itself. Even though melanoma was among the first cancers recognized as a target for practical application of microarray analysis starting in 1996 [24], the transition of gene expression results to diagnostic applications with clinical impact has not been shown yet in MM. Microarray studies produced a plethora of data and have provided useful insights into the molecular biology of melanoma (reviewed in [25]). However, analysis of different histological subtypes of melanoma, expression analysis of mainly melanoma metastases, and lack of homogeneity of patient cohorts hampered the interpretation of these data [25]. Furthermore, routine supply of fresh frozen MM tissue for microarray based assays seems virtually impossible. Also, use of RT-PCR based tests are complicated by the need for pure populations of neoplastic melanoma cells, and has not yet resulted in a breakthrough in melanoma diagnosis or management of melanoma patients. Our defined seven-marker signature was of independent prognostic relevance in two independent patient cohorts. For the practitioner, the assessment of this set of seven IHC markers promises to be a helpful tool to answer the crucial question “whom to treat, and how to treat”, especially in the adjuvant setting after surgical excision of early-stage and localized primary MM (Stage I to IIa). With MTAP, COX-2 and Bcl-X, three markers of the seven-marker signature offer direct therapeutic implications, since the corresponding drugs have already been approved by the FDA.

Currently, in the adjuvant treatment of MM, interferon alpha is the only clinically accepted therapeutic agent providing a significant (recurrence-free) survival benefit for a small but distinct percentage of patients [26]. On account of the serious side effects and the high costs of the therapy, only those patients with a realistic chance to benefit from interferon should receive this treatment. We have recently shown that there is a clear association between MTAP expression in the primary melanoma and melanoma progression and, even more importantly, response to interferon treatment [12], [27]. This gives rise to the hypothesis that interferon response may be correlated with the expression of interferon response genes such as MTAP.

Cyclooxygenase 2 (COX-2) may represent another promising therapeutic target. Cyclooxygenases catalyze the first rate-limiting step in the conversion of arachidonic acid to prostaglandins. COX-2 is expressed in various tumor types and levels of COX-2 expression have been shown to correlate with invasiveness and prognosis in some tumor entities, including epithelial and melanocytic skin cancer [15], [28]. So far the benefit of COX-2-inhibitors has not been studied in the adjuvant treatment of early-stage melanomas to prevent metastasis. In the second-line treatment of advanced metastatic melanoma disease, however, a survival benefit was shown for targeted combined therapy using COX-2 inhibitors and PPARG-agonists for anti-inflammatory treatment together with low-dose metronomic chemotherapy [29]. Considering this observation and the fact that melanoma patients with COX-2-positive primary tumors bear a significantly higher risk of tumor recurrence [15], introduction of COX-2 inhibitors for primary adjuvant treatment of these patients seems obvious.

One additional marker, Bcl-X, has been targeted in preclinical tests and several targeting agents are in the clinical testing phase by now [30]: Bcl-X is related to the anti-apoptotic Bcl-2 protein family. Overexpression of these anti-apoptotic proteins protects cancer cells against death signals of apoptosis. Interestingly, tumors expressing high levels of Bcl-2 or Bcl-X are often found to be resistant to chemotherapeutic agents or radiation therapy [31]. In recent years, non-peptidic cell permeable “small molecule inhibitors” (SMIs) against antiapoptotic proteins like Bcl-2 or Bcl-X have been identified. SMIs inhibit distinct protein-protein interactions by blocking specific binding sites of the target molecule, thus supporting the apoptotic machinery [30]. Inhibition of Bcl-X may exert a synergistic effect with conventional treatments like chemo- or radiation therapy. Regarding MM therapy, this effect would be a decisive therapeutic success.

Presence of CD20-positive B-lymphocytes within or adjacent to MM tissue (Figures 8A–D ) was among the top seven biomarkers. However, the role of B-lymphocyte infiltration in MM is unknown and needs further investigation. The CD20-antigen is known to be an effective therapeutic target in the treatment of patients with CD20-positive B-Cell-Non-Hodgkin-Lymphomas. The monoclonal chimeric antibody Rituximab is indicated for alternative immunotherapy [32]. In MM, several subpopulations - some with stem cell-like characteristics - have been described including one with expression of CD20 (reviewed in [33]). In our study, only very few cases showed infiltrating CD20 positive B-lymphocytes (Figure 2E), comprising a very narrow dynamic range of this marker. However, even a six-marker signature without CD20 was significantly associated with overall and recurrence-free survival (Figures 9A and 9B).

The tumor-suppressor gene phosphatase and tensin homolog (PTEN) is one of the most commonly inactivated genes in human cancer and has been identified as lost or mutated in melanoma [34]. An established consequence of PTEN inactivation is the constitutive aberrant activation of the phosphatidylinositol-3-kinase (PI3K)-signaling pathway that drives uncontrolled cell growth, proliferation, and survival [35]. In general, prediction of PI3K-signaling pathway activation based on PTEN IHC expression status is unfeasible since inactivation of PTEN is achieved by either gene mutation or deletion [34]. Figure S5 shows that some melanoma cell lines show PTEN expression and concomitant activation of the PI3K/AKT cascade. Alterations of the tumor suppressor PTEN have already been linked with disease outcome in patients with MM: Mikhail et al. have shown that loss of nuclear PTEN expression was associated with aggressive tumor behavior [36]. In contrast, we could show that strong cytoplasmic PTEN expression was found only in high risk patients.

Most oncological findings regarding the Wnt/β-Catenin signaling are derived from the analysis of colon, breast and kidney carcinoma [37], [38] where activation of the pathway has been directly implicated in disease pathogenesis. The majority of colorectal carcinomas carry inactivating mutations in the Adenomatous polyposis coli (APC) tumor suppressor which lead to stabilization of β-Catenin, mimicking Wnt stimulation. Additionally, mutations in the β-Catenin gene *CTNNB1* were found in colon cancer leading to the constitutive activation of β-Catenin/LEF/TCF-dependent canonical signaling [39]. However, such mutations are rarely found during melanoma development. In contrast to findings in colon carcinoma and in line with a study by Kuphal et al. [40], we show that β-Catenin protein was basically cytoplasmic in melanomas *in vivo.* Regarding our risk score, loss of cytoplamic β-Catenin expression was associated with worse outcome of melanoma patients. Kuphal and co-workers also demonstrated that the transcriptional activity of β-Catenin regulating expression of β-Catenin target genes was not observed in several melanoma cell lines, suggesting a cell type specific regulation of β-Catenin function [40].

According to the data presented here, the seven-marker signature might serve as a prognostic tool enabling physicians to selectively triage, at the time of diagnosis and initial surgery, the subset of high recurrence risk stage I–II patients for adjuvant therapy. Selective treatment of those patients that are more likely to develop distant metastatic disease could potentially lower the burden of untreatable metastatic melanoma and revolutionize the therapeutic management of MM. Prospective clinical trials are necessary to validate the prognostic and therapeutic value of this seven-marker signature and its benefit for routine clinical assessment of MM. The utility of the algorithm in a practice setting, where full heterogeneous tissue sections are used, is currently being analyzed in a prospective clinical trial at the Department of Dermatology, University of Regensburg, Germany.

## Supporting Information

Figure S1 **Western blot analysis of the nine-marker signature candidates in lysates of melanocytes and human melanoma cell lines.** Cultured cells were lysed in 4°C cold radioimmunoprecipitation assay buffer (RIPA Buffer Set, Boehringer, Mannheim, Germany: 50 mM Tris-HCl, pH 7.5, 150 mM NaCl, 1% Nonidet® P40, 0.5% sodium deoxycholate, 0.1% SDS, 1 Complete™ Protease Inhibitor Cocktail Tablet). Protein extracts (40 µg) were run on 8–15% polyacrylamide gels, transferred to polyvinylidene fluoride (PVDF) membranes (Millipore, Bedford, USA) and visualised by immunoblotting. Human melanoma cell lines*, melanocytes and keratinocytes were provided by Anja K. Bosserhoff, Institute of Pathology, University of Regensburg, Germany. Whole cell lysates for positive controls were provided by Abcam plc, 330 Science Park, Cambridge, CB4 0FL, UK. Whole cell lysates of human HaCaT keratinocytes were provided by the DKFZ, Heidelberg, Germany.
(PDF)

Figure S2 **Immunohistochemical analysis of the seven-marker signature candidates using a microarray with cell pellets of melanocytes and human melanoma cell lines.** In order to characterise the seven-marker signature candidates, melanocytes and melanoma cell lines were trypsinized and embedded in paraffin as a cell pellet. Sections of these cell blocks were stained with antibodies against the seven-marker signature. All immunohistochemical investigations were based on an avidin-biotin peroxidase method with a 3-amino-9-ethylcarbazole (AEC) chromatogen. After antigen retrieval (steam boiler with citrate-buffer, pH 6.0 or with Tris-EDTA-buffer, pH 9.0 for 20 min) immunohistochemistry was carried out applying the ZytoChemPlus HRP Broad Spectrum Kit (Zytomed Systems, Berlin, Germany) according to the manufacturer’s instructions. Cytoplasmic and nuclear markers were visualized with AEC solution (AEC+ High Sensitivity Substrate Chromogen, ready-to-use, DAKO, Glostrup, Denmark). The red color of the AEC substrate chromogen (3-amino-9-ethylcarbazole) is very beneficial to rule out the possibility of a role of endogenous melanin in the observed reactivity. All sections were counterstained with hematoxylin (DAKO). Weblink to slides: http://histodb2.usz.ch/dss/searchURL.php?outputFormat=viewer&category=conference&confHash=-275761229
(PDF)

Figure S3 **Immunohistochemical analysis of the marker heterogeneity of the seven-marker signature candidates, staining a selection of whole slides for each marker.** Besides infiltrating CD20 positive B-Lymphocytes, the staining distribution of the remaining six markers was rather homogenous.
(PDF)

Figure S4 **Bar chart depicting Pearson correlation coefficents between the markers of the signature.**
(PDF)

Figures S5 **Immunohistochemical characterization of two melanoma cell lines regarding the phosphatidylinositol-3-kinase (PI3)/AKT/mTOR cascade.** In order to characterise the melanoma cell lines that were used for our western blot experiments, cells were trypsinized and embedded in paraffin as a cell pellet. Sections of these cell blocks were stained with antibodies against PTEN (Dako, clone 6H2.1, M3627, dilution 1∶200), P-Akt (Ser473) (Abcam, ab8932, dilution 1∶150), and P-S6 ribosomal protein (Ser235/236, Cell Signaling Technology, #2215, dilution 1∶50). PTEN status of prostate cancer cell lines is well known. PC3 cells typically have sustained a homozygous deletion of PTEN and are therefore PTEN negative (Panel A). In contrast LNCaP cells have a deletion of one allele and a mutation of the other PTEN allele (McMenamin ME, et al. (1999) Cancer Res 59∶4291–4296) with consecutive PTEN overexpression (Panel B). Both cell lines typically show activation of the phosphatidylinositol-3-kinase (PI3)/AKT cascade, resulting in expression of P-Akt and P-S6 ribosomal protein. The two melanoma cell lines tested (HTZ 19d and IGR-1), both showed expression of PTEN and activation of the phosphatidylinositol-3-kinase (PI3)/AKT cascade (Panel C&D).
(PDF)

Table S1 **Characterization and comparison of the primary cohort (TMA 1) and the external test cohort (TMA 2).** Reported are the number of counts and the associated percentages for all specimens on the tissue microarrays. CD49d and MLH1 are not contained in the final seven-marker signature and therefore were not analyzed on the external test TMA 2. Missing values are listed as “unknown”.
(XLS)

Table S2 **Properties of the 70 biomarker candidates for malignant melanoma immunohistochemically analyzed in this study.** All antibodies investigated are listed indicating source, dilution, pattern of reactivity and positive control. The described signature was statistically learned by the FDR selection procedure from this pool of 70 biomarkers.
(DOCX)
