# Supplementary figures and images for: A Seven-Marker Signature and Clinical Outcome in Malignant Melanoma: A Large-Scale Tissue-Microarray Study with Two Independent Patient Cohorts
Source: PLoS One. 2012 Jun 7;7(6):e38222. doi: 10.1371/journal.pone.0038222 (PMC3369875; doi:10.1371/journal.pone.0038222)

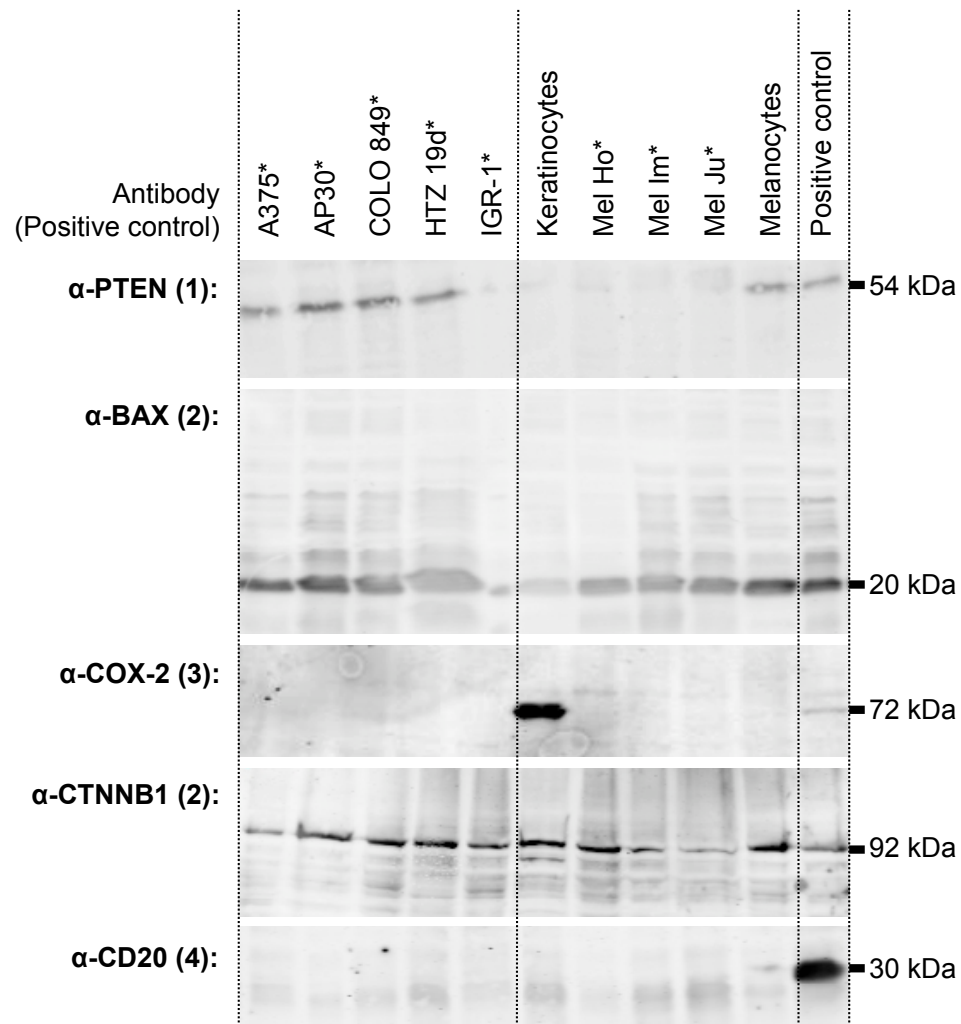

**Positive control:**

- |            |                 |
|------------|-----------------|
| (1) HL-60  | (4) Daudi       |
| (2) HEK293 | (5) Jurkat      |
| (3) HaCaT  | (6) Melanocytes |
- NA, not available

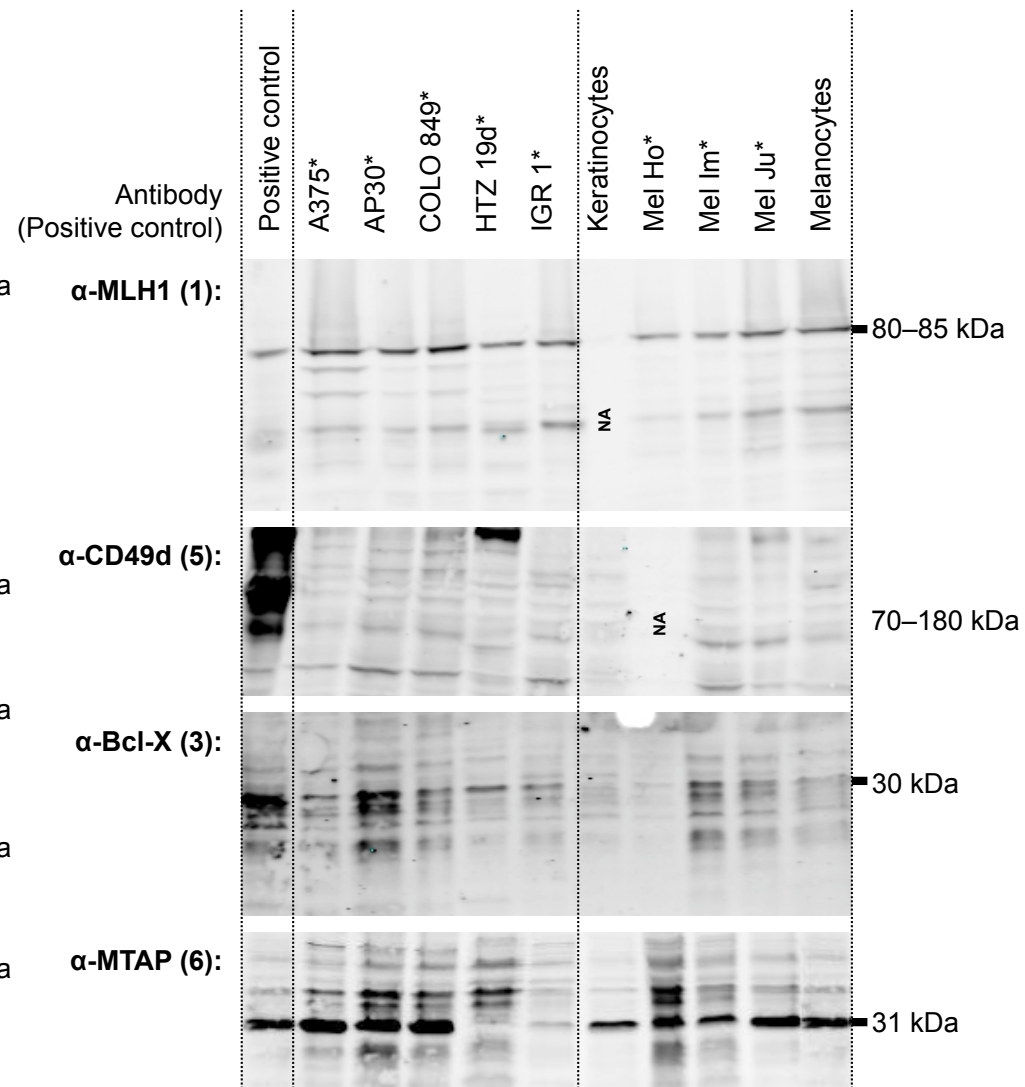

Supplement: Figure S1 — Western blot analysis of the nine-marker signature candidates in lysates of melanocytes and human melanoma cell lines. Cultured cells were lysed in 4°C cold radioimmunoprecipitation assay buffer (RIPA Buffer Set, Boehringer, Mannheim, Germany: 50 mM Tris-HCl, pH 7.5, 150 mM NaCl, 1% Nonidet® P40, 0.5% sodium deoxycholate, 0.1% SDS, 1 Complete™ Protease Inhibitor Cocktail Tablet). Protein extracts (40 µg) were run on 8–15% polyacrylamide gels, transferred to polyvinylidene fluoride (PVDF) membranes (Millipore, Bedford, USA) and visualised by immunoblotting. Human melanoma cell lines*, melanocytes and keratinocytes were provided by Anja K. Bosserhoff, Institute of Pathology, University of Regensburg, Germany. Whole cell lysates for positive controls were provided by Abcam plc, 330 Science Park, Cambridge, CB4 0FL, UK. Whole cell lysates of human HaCaT keratinocytes were provided by the DKFZ, Heidelberg, Germany. (PDF) [file pone.0038222.s001.pdf]

## Correlation between Markers of Signature

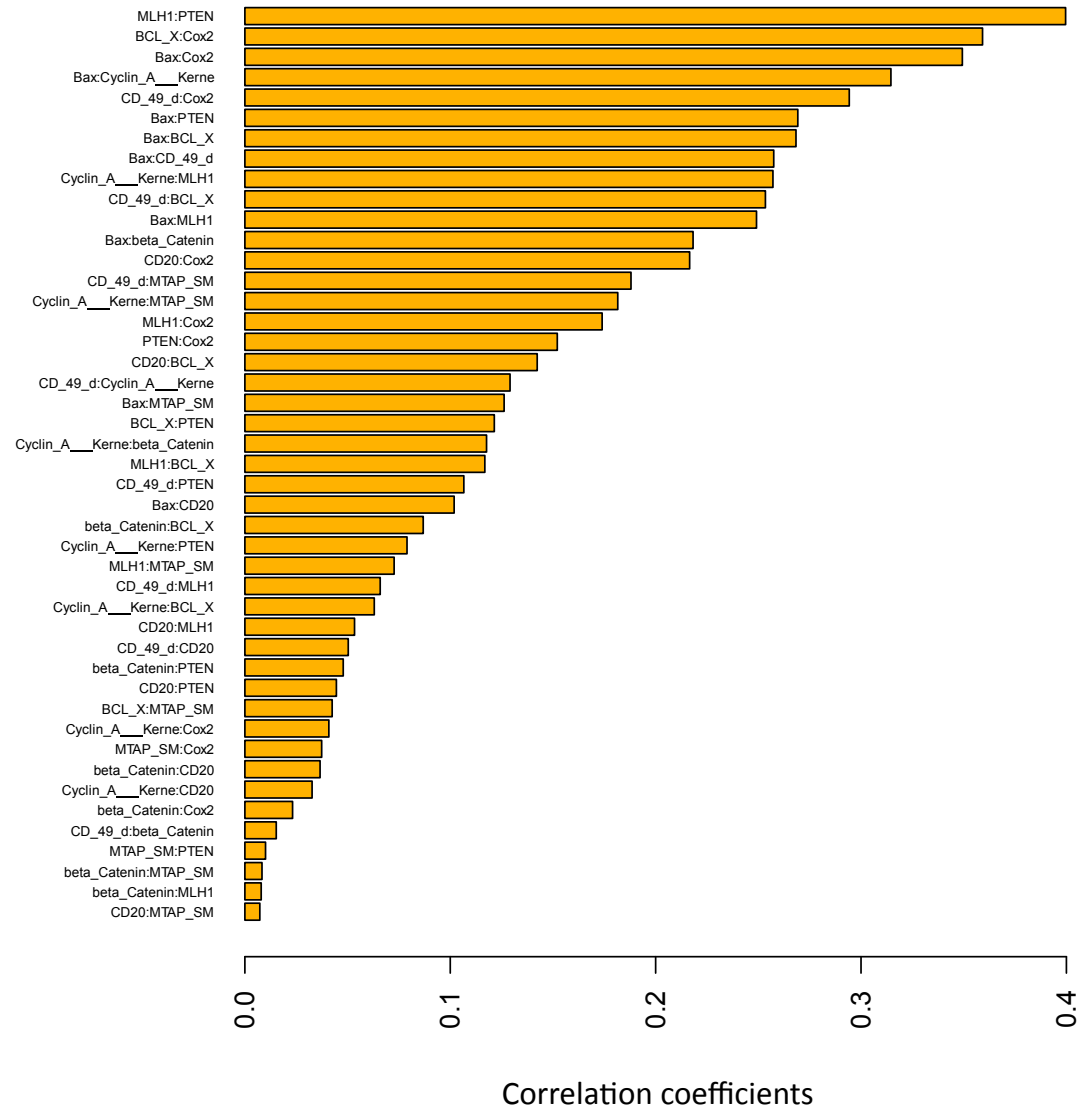

Supplement: Figure S4 — Bar chart depicting Pearson correlation coefficents between the markers of the signature. (PDF) [file pone.0038222.s004.pdf]
