# Supplementary material for: A Seven-Marker Signature and Clinical Outcome in Malignant Melanoma: A Large-Scale Tissue-Microarray Study with Two Independent Patient Cohorts
Source: PLoS One. 2012 Jun 7;7(6):e38222. doi: 10.1371/journal.pone.0038222 (PMC3369875; doi:10.1371/journal.pone.0038222)
Supplement: Figure S2 — Immunohistochemical analysis of the seven-marker signature candidates using a microarray with cell pellets of melanocytes and human melanoma cell lines. In order to characterise the seven-marker signature candidates, melanocytes and melanoma cell lines were trypsinized and embedded in paraffin as a cell pellet. Sections of these cell blocks were stained with antibodies against the seven-marker signature. All immunohistochemical investigations were based on an avidin-biotin peroxidase method with a 3-amino-9-ethylcarbazole (AEC) chromatogen. After antigen retrieval (steam boiler with citrate-buffer, pH 6.0 or with Tris-EDTA-buffer, pH 9.0 for 20 min) immunohistochemistry was carried out applying the ZytoChemPlus HRP Broad Spectrum Kit (Zytomed Systems, Berlin, Germany) according to the manufacturer’s instructions. Cytoplasmic and nuclear markers were visualized with AEC solution (AEC+ High Sensitivity Substrate Chromogen, ready-to-use, DAKO, Glostrup, Denmark). The red color of the AEC substrate chromogen (3-amino-9-ethylcarbazole) is very beneficial to rule out the possibility of a role of endogenous melanin in the observed reactivity. All sections were counterstained with hematoxylin (DAKO). Weblink to slides: http://histodb2.usz.ch/dss/searchURL.php?outputFormat=viewer&category=conference&confHash=-275761229 (PDF) [file pone.0038222.s002.pdf]

Block diagram of cell pellet microarray

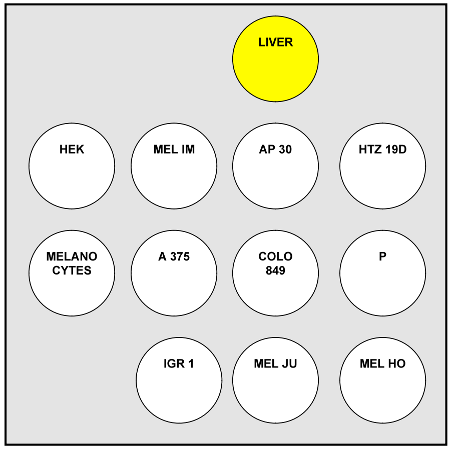

Catenin-beta

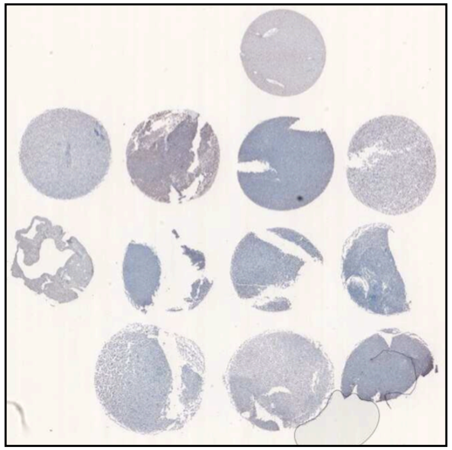

Bax

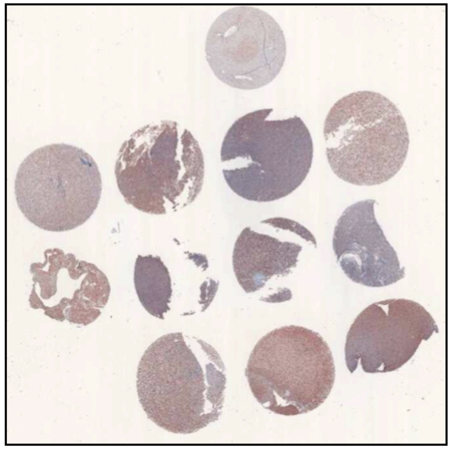

Bcl-X

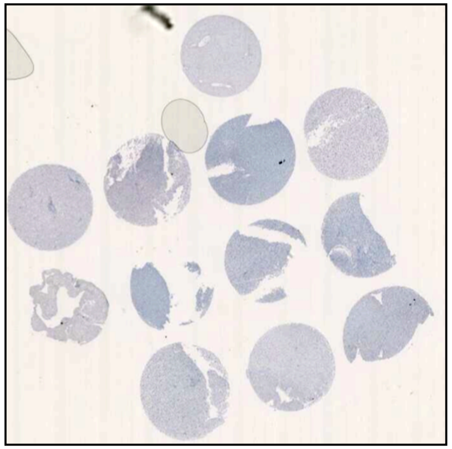

CD20

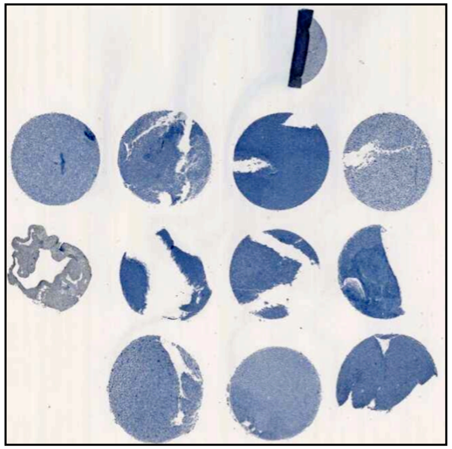

COX-2

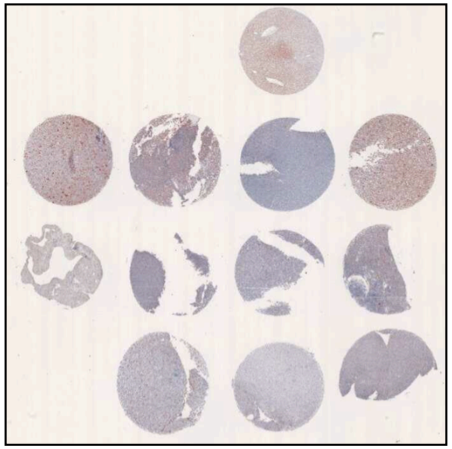

PTEN

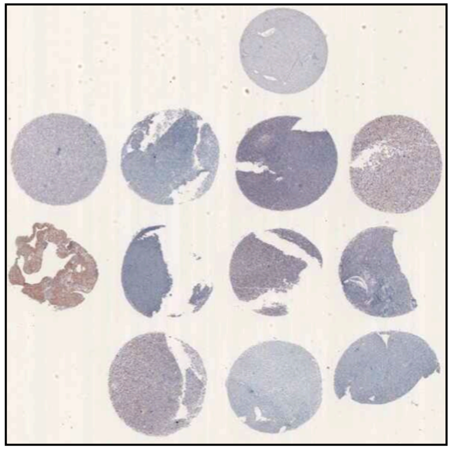

MTAP

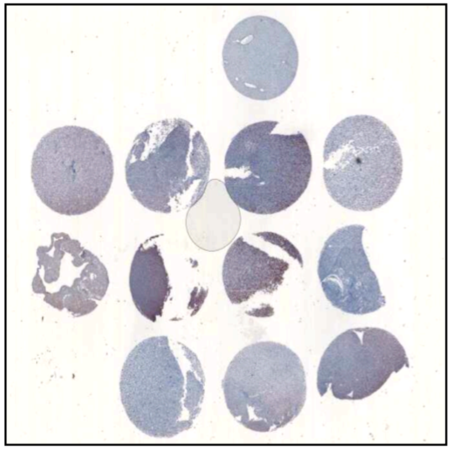

Weblink to slides:
http://histodb2.usz.ch/dss/searchURL.php?outputFormat=viewer&category=conference&confHash=-275761229
