# Supplementary material for: A Seven-Marker Signature and Clinical Outcome in Malignant Melanoma: A Large-Scale Tissue-Microarray Study with Two Independent Patient Cohorts
Source: PLoS One. 2012 Jun 7;7(6):e38222. doi: 10.1371/journal.pone.0038222 (PMC3369875; doi:10.1371/journal.pone.0038222)

A Nodular malignant melanoma, Breslow 1.7 mm, Clark level IV (Example #1)

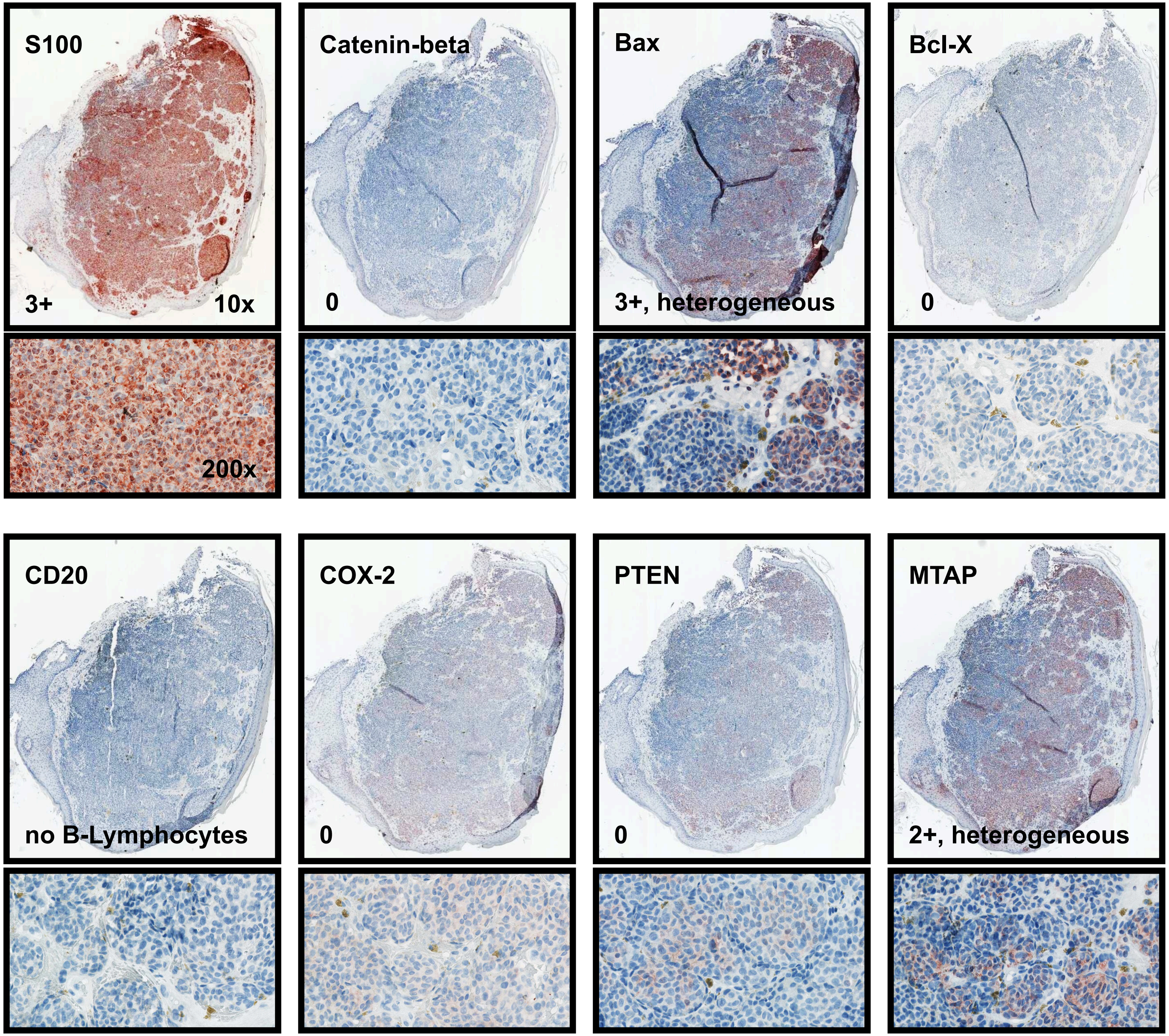

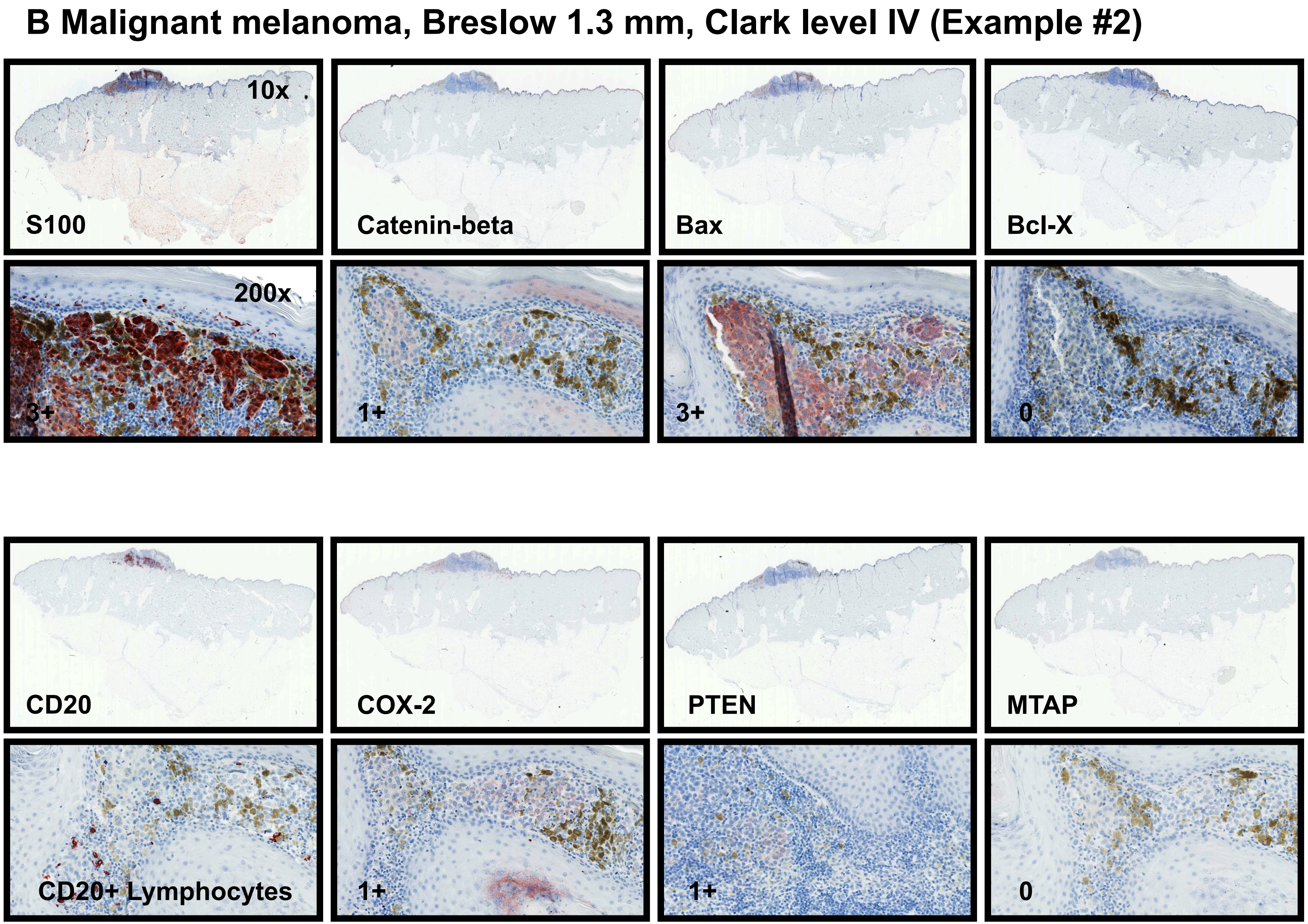
B Malignant melanoma, Breslow 1.3 mm, Clark level IV (Example #2)
10x
S100
Catenin-beta
Bax
Bcl-X
200x
3+
1+
3+
0
CD20
COX-2
PTEN
MTAP
CD20+ Lymphocytes
1+
1+
0

C Superficial spreading melanoma, Breslow 0.15 mm, Clark level II (Example #3)

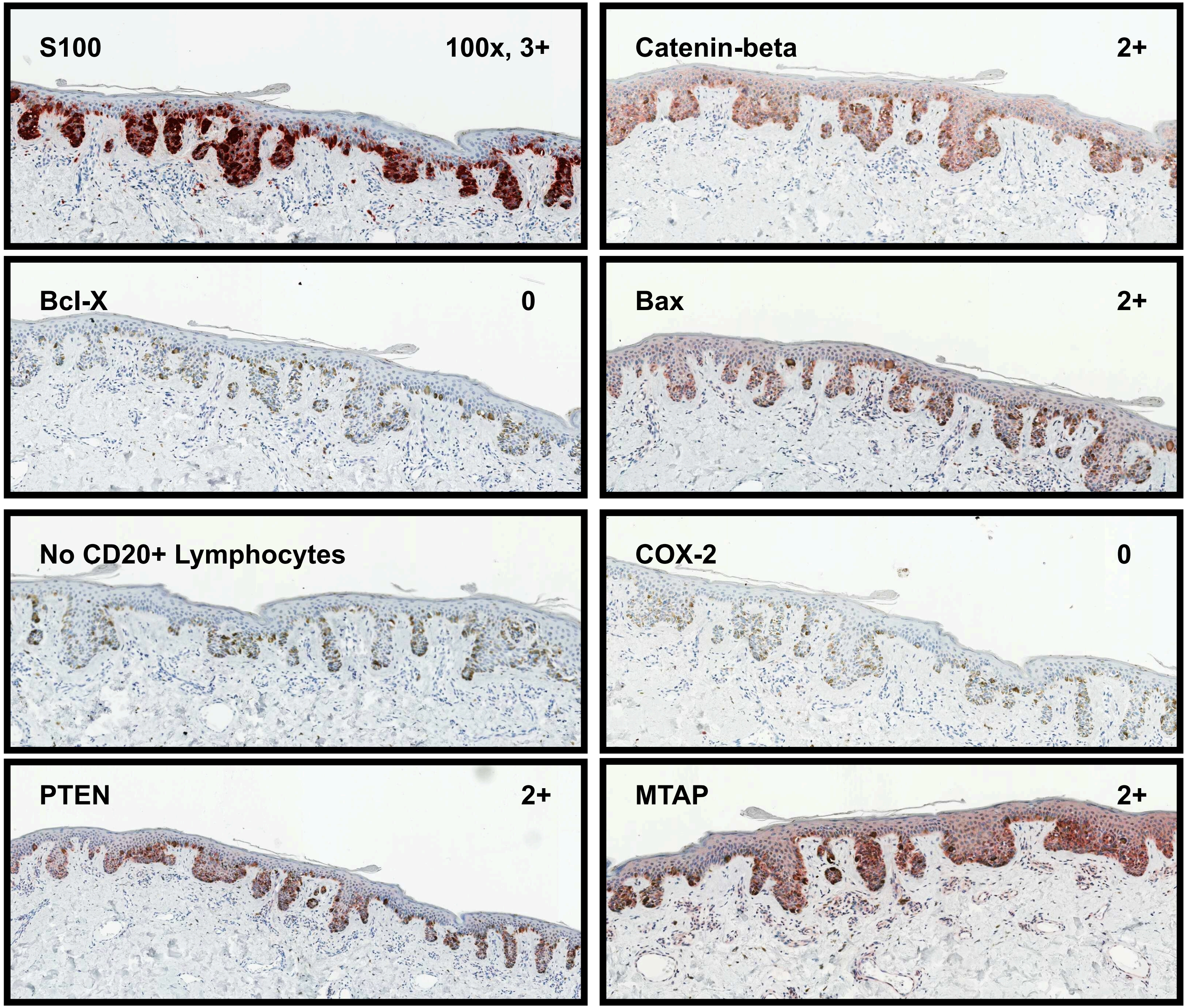

D Lentigo maligna melanoma, Breslow 1.2 mm, Clark level IV (Example #4)

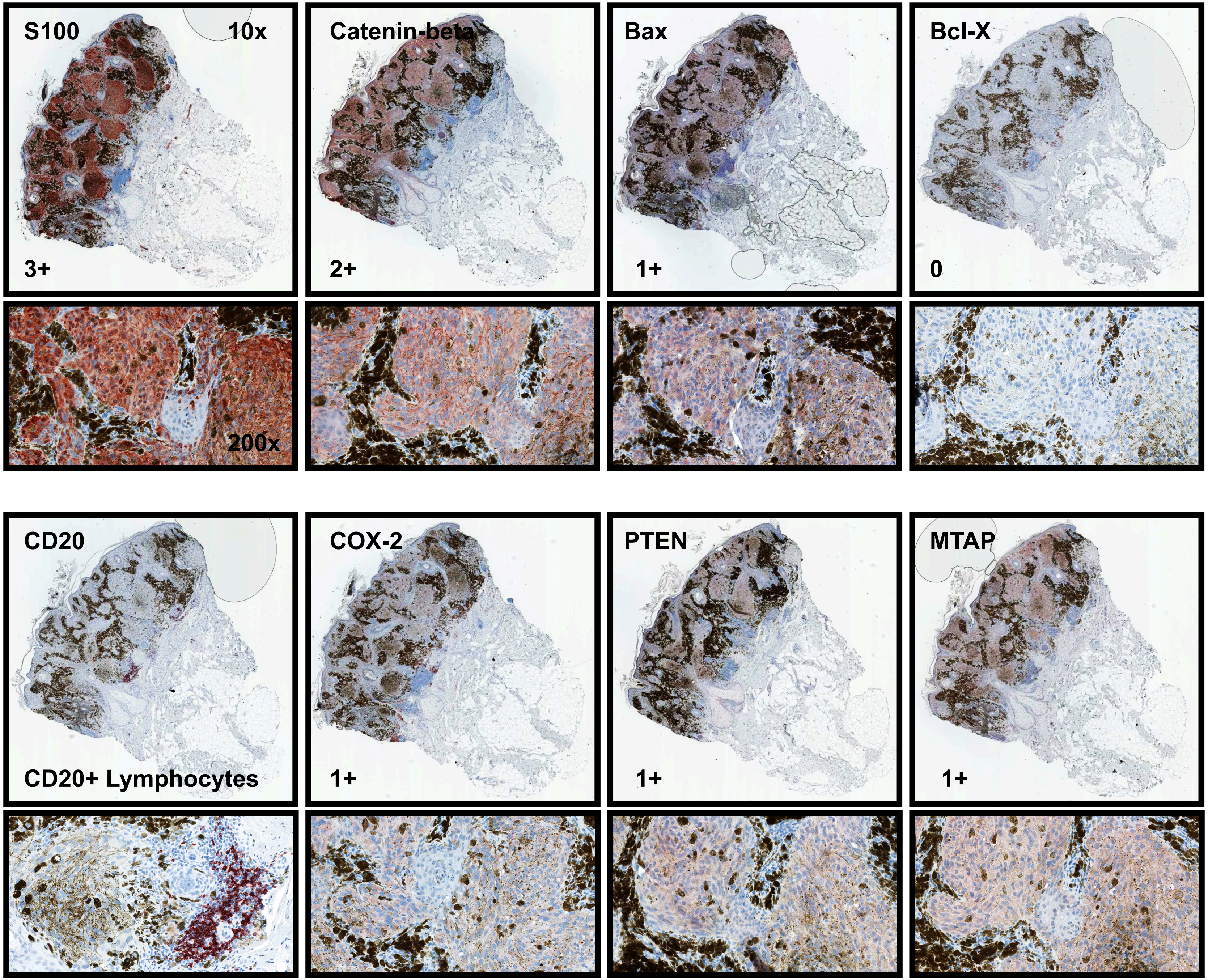

Supplement: Figure S3 — Immunohistochemical analysis of the marker heterogeneity of the seven-marker signature candidates, staining a selection of whole slides for each marker. Besides infiltrating CD20 positive B-Lymphocytes, the staining distribution of the remaining six markers was rather homogenous. (PDF) [file pone.0038222.s003.pdf]
