# Supplementary material for: A Seven-Marker Signature and Clinical Outcome in Malignant Melanoma: A Large-Scale Tissue-Microarray Study with Two Independent Patient Cohorts
Source: PLoS One. 2012 Jun 7;7(6):e38222. doi: 10.1371/journal.pone.0038222 (PMC3369875; doi:10.1371/journal.pone.0038222)
Supplement: Figures S5 — Immunohistochemical characterization of two melanoma cell lines regarding the phosphatidylinositol-3-kinase (PI3)/AKT/mTOR cascade. In order to characterise the melanoma cell lines that were used for our western blot experiments, cells were trypsinized and embedded in paraffin as a cell pellet. Sections of these cell blocks were stained with antibodies against PTEN (Dako, clone 6H2.1, M3627, dilution 1∶200), P-Akt (Ser473) (Abcam, ab8932, dilution 1∶150), and P-S6 ribosomal protein (Ser235/236, Cell Signaling Technology, #2215, dilution 1∶50). PTEN status of prostate cancer cell lines is well known. PC3 cells typically have sustained a homozygous deletion of PTEN and are therefore PTEN negative (Panel A). In contrast LNCaP cells have a deletion of one allele and a mutation of the other PTEN allele (McMenamin ME, et al. (1999) Cancer Res 59∶4291–4296) with consecutive PTEN overexpression (Panel B). Both cell lines typically show activation of the phosphatidylinositol-3-kinase (PI3)/AKT cascade, resulting in expression of P-Akt and P-S6 ribosomal protein. The two melanoma cell lines tested (HTZ 19d and IGR-1), both showed expression of PTEN and activation of the phosphatidylinositol-3-kinase (PI3)/AKT cascade (Panel C&D). (PDF) [file pone.0038222.s005.pdf]

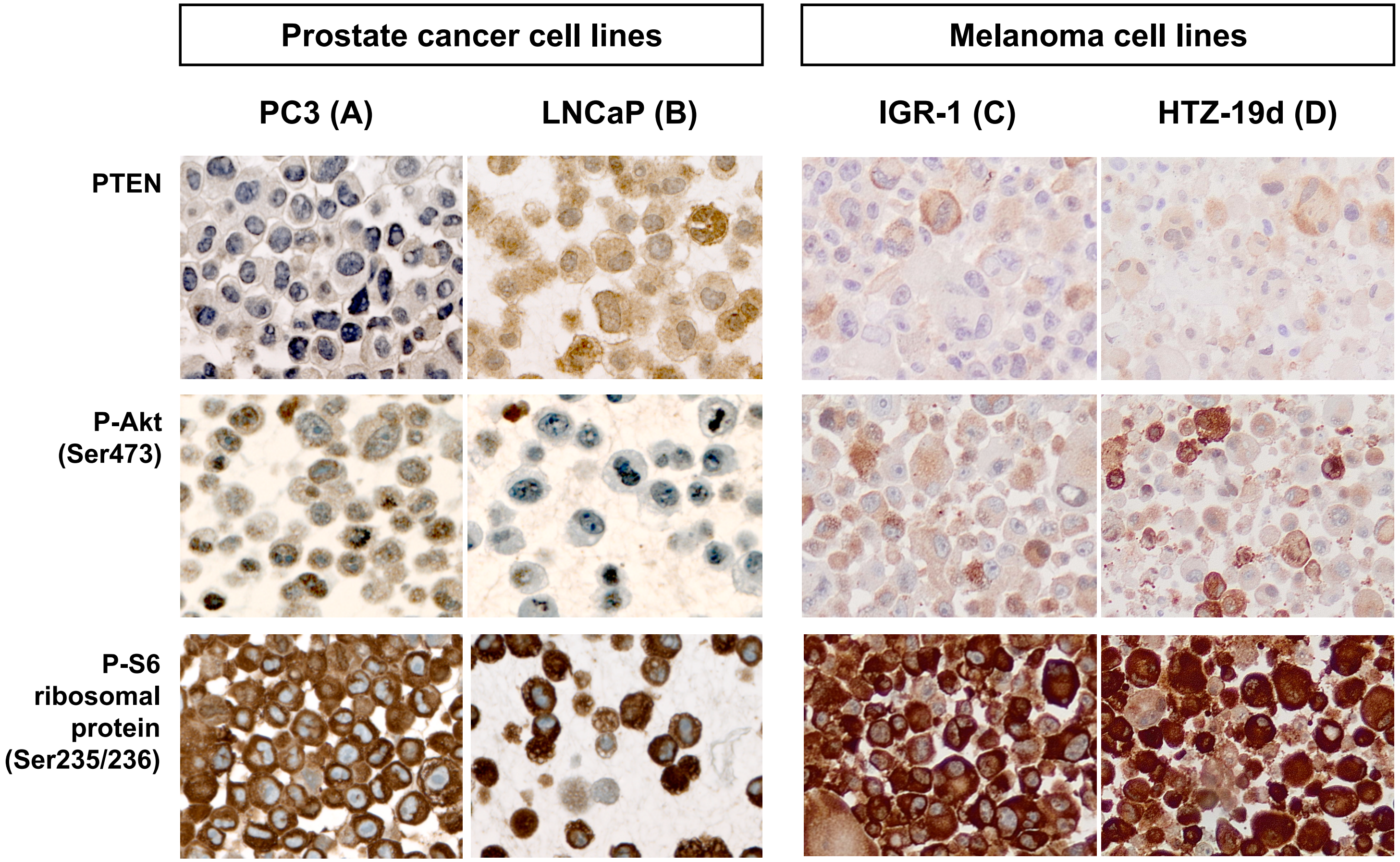
Prostate cancer cell lines
Melanoma cell lines
PC3 (A)
LNCaP (B)
IGR-1 (C)
HTZ-19d (D)
PTEN
P-Akt (Ser473)
P-S6 ribosomal protein (Ser235/236)
