# Supplementary material for: A Seven-Marker Signature and Clinical Outcome in Malignant Melanoma: A Large-Scale Tissue-Microarray Study with Two Independent Patient Cohorts
Source: PLoS One. 2012 Jun 7;7(6):e38222. doi: 10.1371/journal.pone.0038222 (PMC3369875; doi:10.1371/journal.pone.0038222)
Supplement: Table S2 — Properties of the 70 biomarker candidates for malignant melanoma immunohistochemically analyzed in this study. All antibodies investigated are listed indicating source, dilution, pattern of reactivity and positive control. The described signature was statistically learned by the FDR selection procedure from this pool of 70 biomarkers. (DOCX) [file pone.0038222.s007.docx]

| **Protein**  **Name** | **HUGO: Approved Symbol** | **Functional Group** | **Source** | **Catalog**  **Number** | **Clone** | **Dilution** | **Cellular**  **Localization** | **Positive Control** |
| --- | --- | --- | --- | --- | --- | --- | --- | --- |
| Akt 3 | AKT3 | Signaling, Apoptosis | Abgent | AP7030a | Rabbit,  Polyclonal | 1:100 | Cytoplasmic | Breast cancer |
| Phospho-Akt (Thr308) | Phospho-AKT3 | Signaling, Apoptosis | Cell Signaling | 9266 | Rabbit,  244F9H2 | 1:10 | Cytoplasmic | Breast cancer |
| Bax | BAX | Apoptosis | Cell Signaling | 2774 | Rabbit,  polyclonal | 1:10 | Cytoplasmic | Lung cancer |
| Bcl2 | BCL2 | Apoptosis | Dako | M 0887 | Mouse,  124 | 1:100 | Cytoplasmic | Kidney |
| Bcl-X | BCL2L1 | Apoptosis | Diagnostic  Biosystems | Mob 248 | Mouse,  2H12 | 1:10 | Cytoplasmic,  cell membrane | Tonsil |
| Bcl2L1 | BCL2L1 | Apoptosis | Abcam | ab17834 | Mouse,  SPM165 | 1:200 | perinuclear | Melanoma |
| BMI1 | BMI1 | Transcription | Abgent | AP2513c | Rabbit,  polyclonal | 1:25 | Cytoplasmic | Breast cancer |
| B-Raf | BRAF | Signaling, Apoptosis | Epitomics | 1647-1 | Rabbit,  EP152Y | 1:50 | Cytoplasmic | Prostate cancer |
| E-Cadherin | CDH1 | Cell-cell contact | Chemicon | IHCR2123-6 | Mouse, 36B5 | r-t-u | Cell membrane | Breast cancer |
| P-Cadherin | CDH3 | Cell-cell contact | BD Biosciences | 610227 | Mouse,  56 | 1:20 | Cell membrane | Placenta |
| β-Catenin | CTNNB1 | Cell-cell contact | Cell Signaling | 9562 | Rabbit, polyclonal | 1:250 | Cytoplasmic, nuclear,  cell membrane | Breast cancer |
| **Protein**  **Name** | **HUGO: Approved Symbol** | **Functional Group** | **Source** | **Catalog Number** | **Clone** | **Dilution** | **Cellular**  **Localization** | **Positive Control** |
| Phospho-β-Catenin | Phospho-  CTNNB1 | Cell-cell contact | Cell Signaling | 9561 | Rabbit, polyclonal | 1:50 | Nuclear | Breast cancer |
| Caveolin | CAV1 | Cell-cell contact | BD  Biosciences | 610059 | Rabbit, polyclonal | 1:1000 | Cell membrane | Placenta |
| CD20 | MS4A1 | Differentiation,  Stem cell marker cand. | Zytomed | BMS003 | Mouse,  L26 | 1:1 | Cell membrane cytoplasmic | Tonsil |
| CD44 | CD44 | Cell-cell contact,  Stem cell marker cand. | Lab Vision | MS-668-R7 | Mouse,  156-3C11 | 1:2000 | Cell membrane | Tonsil |
| CD49d | ITGA4 | Cell-cell contact,  Stem cell marker cand. | Acris | AP00455PU-N | Rabbit,  polyclonal | 1:50 | Cell membrane | Breast cancer |
| CD 117  (c-kit) | KIT | Proliferation,  Stem cell marker cand. | Dako | A4502 | Rabbit,  polyclonal | 1:600 | Cell membrane cytoplasmic | Colorectal cancer |
| CD 166 | ALCAM | Cell-cell contact,  Stem cell marker cand. | Abcam | ab49496 | Mouse,  MOG/07 | 1:50 | Cell membrane | Prostate cancer |
| CD 171 | L1CAM | Cell-cell contact | Sigma | HPA005830 | Rabbit | 1:350 | Cytoplasmic | Breast cancer |
| CDK2 | CDK2 | Cell cycle | Thermo Scientific | MS-617-P1 | Mouse, 2B6 + 8D4 | 1:200 | Cytoplasmic, nuclear | Tonsil |
| c-Myc | MYC | Cell cycle, Proliferation | Santa Cruz | sc-70463 | Mouse, monoclonal | 1:500 | Cytoplasmic, nuclear | Colorectal cancer |
| Cox-2 | MT-CO2 | Metabolism | Cayman | 160112 | Mouse,  monoclonal | 1:200 | Cytoplasmic | Colorectal cancer |
| **Protein**  **Name** | **HUGO: Approved Symbol** | **Functional Group** | **Source** | **Catalog**  **Number** | **Clone** | **Dilution** | **Cellular**  **Localization** | **Positive Control** |
| CXCR4 | CXCR4 | Cell-cell contact,  Stem cell marker cand. | Acris | SP4106P | Rabbit,  polyclonal | 1:750 | Cytoplasmic,  cell membrane | Breast cancer |
| Cyclin A | CCNA1 | Cell cycle | Novo Castra | NCL-CYCLIN A | Mouse,  6E6 | 1:50 | nuclear | Tonsil |
| Cyclin D1 | CCND1 | Cell cycle | Novo Castra | NCL-CYCLIN D1 | Mouse,  DCS-6 | 1:20 | Nuclear, cytoplasmic | Breast cancer |
| Eph B2 | EPHB2 | Cell-cell contact | Abcam | ab5418 | Rabbit,  polyclonal | 1:100 | Cell membrane  cytoplasmic | Breast cancer |
| ephrin-B2 | EFNB2 | Cell-cell contact | Santa Cruz | sc-15397 | Rabbit, polyclonal | 1:50 | Cell membrane  cytoplasmic | Melanoma |
| Ezrin | EZR | Signaling | Abcam | ab4069 | Mouse,  3C12 | 1:100 | Cell membrane | Lung Cancer |
| Fas | FAS | Apoptosis | Cell Signaling | 4233 | Rabbit,  C18C12 | 1:10 | Cytoplasmic | Colorectal cancer |
| FZD-7 | FZD7 | Signaling | Acris | SP4149P | Rabbit,  polyclonal | 1:250 | Cytoplasmic | Placenta |
| Glut-1 | SLC2A1 | Metabolism | Acris | AM00389PU-N | Mouse, SPM498 | 1:200 | Cell membrane | Breast cancer |
| HIF-1α | HIF1A | Metabolism | R+D | MAB1935 | Mouse,  241812 | 1:20 | Cytoplasmic, nuclear | Breast cancer |
| Anti-Melanosome HMB45 | -/- | Differentiation | Dako | M0634 | Mouse, HMB45 | 1:50 | Cytoplasmic | Melanoma |
| **Protein**  **Name** | **HUGO: Approved Symbol** | **Functional Group** | **Source** | **Catalog Number** | **Clone** | **Dilution** | **Cellular**  **Localization** | **Positive Control** |
| IGF-2 | IGF2 | Proliferation | Abcam | ab9574 | Rabbit,  polyclonal | 1:200 | Cytoplasmic | Placenta |
| iNOS | ISYNA1 | Metabolism | Abcam | ab53769 | Rabbit,  polyclonal | 1:200 | Cytoplasmic | Lung cancer |
| Ki-67 | MKI67 | Cell cylce, Proliferation | Dako | M 7240 | Mouse,  MIB-1 | 1:100 | Nuclear | Skin |
| MHC 1 | MYH11 | Cell-cell contact | Abcam | ab682 | Mouse,  3F8 | 1:100 | Cell membrane | Kidney |
| Melan A | MLANA | Differentiation | Novo Castra | NCL-MelanA | Mouse,  A103 | 1:50 | Cytoplasmic | Skin |
| MITF | MITF | Differentiation | Dako | M3621 | Mouse,  D5 | 1:50 | Nuclear | Melanoma |
| MLH 1 | MLH1 | DNA repair | BD Pharmingen | 551091 | Mouse,  G168-15 | 1:50 | Nuclear, cytoplasmic | Colorectal cancer |
| MSH2 | MSH2 | DNA repair | Calbiochem | NA27 | Mouse,  FE11 | 1:40 | Nuclear, cytoplasmic | Colorectal cancer |
| MTAP | MTAP | Metabolism | ProteinTechGroup | 11475-1-AP | Rabbit,  polyclonal | 1:500 | Cytoplasmic | Breast cancer |
| MTSS1 | MTSS1 | Cytoskeletal remodeling | Abnova | H00009788-M01 | Mouse, [2G9](http://www.abnova.com/products/products_detail.asp?Catalog_id=H00009788-M01) | 1:500 | Nuclear, cytoplasmic | Colorectal cancer |
| MUM1p | IRF4 | Transcription | Santa Cruz | sc-56713 | Mouse,  monoclonal | 1:10 | Nuclear, cytoplasmic | Breast cancer |
| **Protein**  **Name** | **HUGO: Approved Symbol** | **Functional Group** | **Source** | **Catalog**  **Number** | **Clone** | **Dilution** | **Cellular**  **Localization** | **Positive Control** |
| NF-κВ | RELA | Transcription | Santa Cruz | sc-8008 | Mouse,  F-6 | 1:500 | Nuclear, cytoplasmic | Breast cancer |
| N-Ras | NRAS | Signaling | Santa Cruz | sc-31 | Mouse,  F155 | 1:50 | Cytoplasmic | Lymph node |
| p14 | CDKN2A | Cell cycle | Cell Signaling | 2407 | Mouse, 4C6/4 | 1:10 | Nuclear, cytoplasmic | Breast cancer |
| p15 | CDKN2B | Cell cycle | Acris | DM379 | Mouse,  15P06 | 1:25 | Nuclear | Colorectal cancer |
| p16 | CDKN2A | Cell cycle | Santa Cruz | sc-1661 | Mouse,  F-12 | 1:50 | Nuclear | Colorectal cancer |
| p21 | CDKN1A | Cell cycle | Dako | M 7202 | Mouse,  SX118 | 1:50 | Nuclear | Colorectal cancer |
| p27 | CDKN1B | Cell cycle | Dako | M 7203 | Mouse,  SX53G8 | 1:50 | Nuclear | Lymph node |
| p53 | TP53 | Cell cycle, Apoptosis | Dako | M 7001 | Mouse,  DO-7 | 1:25 | Nuclear, cytoplasmic | Breast cancer |
| p75 (NGFR) | NGFR | Differentiation | Abcam | ab52987 | Rabbit,  EP1039Y | 1:100 | Cell membrane | Placenta |
| PGF | PGF | Proliferation | ProteinTechGroup | 10642-1-AP | Rabbit,  polyclonal | 1:50 | Cytoplasmic | Breast cancer |
| PMP2 | PMP2 | Differentiation | ProteinTechGroup | 12717-1-AP | Rabbit,  polyclonal | 1:100 | Cytoplasmic | Glioma |
| **Protein**  **Name** | **HUGO: Approved Symbol** | **Functional Group** | **Source** | **Catalog Number** | **Clone** | **Dilution** | **Cellular**  **Localization** | **Positive Control** |
| PPAR α | PPARA | Signaling | Abcam | ab8934 | Rabbit,  polyclonal | 1:500 | Cytoplasmic, nuclear | Breast cancer |
| PTEN | PTEN | Signaling | Cell Signaling | 9559 | Rabbit,  138G6 | 1:100 | Cytoplasmic | Colorectal cancer |
| Rb | RB1 | Cell cycle | Calbiochem | OP66 | Mouse,  LM95.1 | 1:50 | Nuclear | Colorectal cancer |
| Phospho-Rb | Phospho-RB1 | Cell cycle | Cell Signaling | 9308 | Rabbit,  polyclonal | 1:50 | Nuclear | Colorectal cancer |
| Ro-52 | TRIM21 | Autoimmunology | Santa Cruz | sc-25351 | Mouse,  D-12 | 1:200 | Nuclear | n. d. |
| Survivin | BIRC5 | Apoptosis | Cell Signaling | 2808 | Rabbit,  71G4 | 1:100 | Nuclear | Colorectal cancer |
| SKP2 | SKP2 | Cell cycle | Zytomed | 519-13884 | Rabbit,  polyclonal | 1:200 | Nuclear, cytoplasmic | Prostate cancer |
| STAT1 | STAT1 | Signaling | Cell Signaling | 9175 | Rabbit,  42H3 | 1:200 | Cytoplasmic, nuclear | Colorectal cancer |
| Phospho-STAT1(S727) |  | Signaling | Abcam | ab47754 | Rabbit,  polyclonal | 1:100 | Cytoplasmic, nuclear | Breast cancer |
| S1P1 | S1PR1 | Cell cycle | Cayman | 10005228 | Rabbit,  polyclonal | 1:50 | Cytoplasmic | Breast cancer |
| TGF-β1 | TGFB1 | Proliferation | Zytomed | 520-11264 | Rabbit,  polyclonal | 1:25 | Cytoplasmic, cell membrane | Colorectal cancer |
| **Protein**  **Name** | **HUGO: Approved Symbol** | **Functional Group** | **Source** | **Catalog Number** | **Clone** | **Dilution** | **Cellular**  **Localization** | **Positive Control** |
| Topoisomerase IIα | TOP2A | DNA repair | Dako | M7186 | Mouse,  Ki-S1 | 1:100 | Nuclear, cytoplasmic | Placenta |
| VEGFR-2 | KDR | Proliferation | Cell Signaling | 2479 | Rabbit,  55B11 | 1:200 | Cytoplasmic | Colorectal cancer |
| XIAP (Birc4) | XIAP | Apoptosis | Lifespan | LS-C43112 | Rabbit,  polyclonal | 1:1000 | Cytoplasmic | Placenta |

**Supplementary Table S2**
